# The Anabranch Framework for the Ruralization of Health Professional Education

**DOI:** 10.3390/healthcare14030406

**Published:** 2026-02-05

**Authors:** Debra Jones, Annemarie Hennessy, Mariah Goldsworthy, Xiang-Yu Hou, Sandra Thompson, Hannah Dean, Kazuma Honda, Danielle Minnis, Charlene Noye, Tracy Robinson, Wendy Gleeson, Reakeeta Smallwood, Aliza Lord, Brendan McCormack, Danielle White

**Affiliations:** 1 Sydney Nursing School, Faculty of Medicine and Health, University of Sydney, Sydney, NSW 2006, Australia; mariah.goldsworthy@sydney.edu.au (M.G.); xiang-yu.hou@sydney.edu.au (X.-Y.H.); hannah.dean@sydney.edu.au (H.D.); kazuma.honda@sydney.edu.au (K.H.); danielle.minnis@sydney.edu.au (D.M.); charlene.noye@sydney.edu.au (C.N.); tracy.robinson@sydney.edu.au (T.R.); reakeeta.smallwood@sydney.edu.au (R.S.); aliza.lord@sydney.edu.au (A.L.); brendan.mccormack@sydney.edu.au (B.M.); danielle.white@sydney.edu.au (D.W.); 2 Faculty of Medicine and Health, University of Sydney, Sydney, NSW 2006, Australia; annemarie.hennessy@sydney.edu.au; 3 Western Australia Centre for Rural Health, The University of Western Australia, Geraldton, WA 6530, Australia; sandra.thompson@uwa.edu.au; 4 Far West Local Health District, New South Wales Health, Broken Hill, NSW 2880, Australia; wendy.gleeson@health.nsw.gov.au

**Keywords:** anabranch framework, colonization, health equity, health professional education, health workforce, rural healthcare, ruralization, University Department of Rural Health, urbanization

## Abstract

**Highlights:**

**What are the main findings?**

- Health professional education is routinely dominated by urban-centric and colonizing worldviews that act to deter practice uptake in rural, remote, and First Nations communities.
- The lack of rural, remote, and First Nations theory, pedagogy, practice, and connectivity in health professional education further marginalizes and stigmatizes these populations, places, and healthcare provision, undermining health equity attainment.

**What are the implications of the main findings?**

- Insight into the challenges confronted by the Australian University Departments of Rural Health in the ruralization of health professional education and practice in achieving substantive health and health workforce outcomes.
- High-level strategic guidance to inform the ruralization of health professional education towards equity-orientated healthcare and the development of a rural-literate, competent, and committed health workforce.

**Abstract:**

**Background/Objective:** The quality of care afforded to rural, remote, and First Nations Peoples is dependent on access to a health workforce with the capacity to contextualize healthcare and practice to the needs and expectations of these populations. In Australia, the lack of representation of rural health in undergraduate and post graduate health professional education undermines this preparedness and consideration of rural practice uptake and longevity, compounding the inequities confronted by 7 million Australians residing in these locations. Urgent educational reforms are required to address this omission, the deficit discourses used to characterize rural healthcare, and the persistent health workforce shortages experienced. This paper presents the Anabranch Framework for the Ruralization of Health Professional Education, a high-level strategy to transform rural healthcare provision, professional practice, and health workforce outcomes. **Methods:** The framework was developed through an iterative process involving a series of systematic steps. The process included the following: individual and group critical dialogues with internal academic educators, external health service leaders, metropolitan academic allies, and leaders of other rural health academic departments; an internal review of empirical studies of relevance to the ruralization of health professional education and practice; the visualization of a place-based framework; the academic conceptualization of the framework; and further critical dialogues to test the framework’s face validity. **Results:** The Anabranch Framework comprises four inter-related rural domains: theories, pedagogies, practices, and connectivity; four constructs: knowledge acquisition and generation, immersion in rural curriculum, knowledge translation and sharing, and relational practice; and two structural elements: spiraled and scaffolded curriculum and duration and the quality of rural placement and practice. **Conclusions:** The Anabranch Framework is a high-level strategy to ruralize health professional worldviews, advance rural person-centered practice, enable a deeper understanding of rural places and the development of an equity-orientated, sustainable and rural-literate health workforce.

## 1. Introduction

Health professional workforce shortages and the resultant health inequities confronting rural and remote (referred to as rural throughout this paper) and First Nations populations are a pervasive challenge globally for government agencies, policy makers, health systems, and the communities and populations they serve [1]. The nature of the education and training health professionals receive is critical in informing and influencing the consideration and uptake of practice in these contexts [2]. Internationally, health practitioners can be required to work in rural settings in response to financial support provided to them by governments to obtain their degrees. In Australia, there are limited mandates that obligate universities to integrate rural health into their health degrees and direct health professionals to practice in these locations post registration. As a result, health professionals have choices about where they practice, the health systems they work in, and the populations they choose to serve. This signposts the importance of challenging the current status quo in higher education towards the ruralization of health professional education and training to address the persistent health inequities and allied health, medicine, and nursing workforce shortages experienced by the 7 million people residing in these locations [2,3,4]. This situation prompted a deep reflection and reconsideration of what is needed to educate, train, and sustain a health workforce that is prepared to work rurally, committed to health equity attainment for rural and First Nations people, and capable of tailoring their care to the needs of these populations. This process resulted in the development of an educational philosophy and approach which revolutionizes the education and training required to develop a competent and dedicated rural Australian health workforce.

### 1.1. Rural Healthcare and Health Professional Education in Australia

The provision of quality rural healthcare is reliant on access to health professionals with the capacity to contextualize their professional practice and healthcare provision to the needs and expectations of rural and First Nations populations [2,4,5]. The education, placement, and practice experiences afforded to health professionals influence their worldviews, consideration and preparedness for rural practice, and the acquisition of the attributes which enable them to respond to the complex policy, political, geographical, social, economic, educational, environmental, and cultural challenges that determine healthcare access and rural health outcomes [1,2,5]. The delivery of ruralized health professional education (HPE) in Australia can be challenging when universities deliver outdated, static, standardized, urban-centric, and colonized curricula that produce ill-equipped graduates to work in these contexts [2,4,5,6].

HPE can be situated with educators with limited to no authentic experience of rural and First Nations healthcare. This education can be delivered in environments dominated by urban-centric and colonizing health and health workforce policy, higher education institutions, health systems, models of care, and clinical practice expectations [1,2,6]. These factors contribute to health professionals failing to recognize and respond to ‘the divergent physical, human resources, and distinctive healthcare needs of rural and remote environments’ [2] (p. 18). The detrimental consequences associated with contemporary HPE are evidenced in protracted rural health workforce shortages [3], institutional and interpersonal racism towards First Nations Peoples [7], the under-representation of First Nations Peoples in Australia’s health workforce [8], and the dominance of hospital-orientated care that weakens health professional capacity to deliver comprehensive public and primary healthcare services [9]. Rural and First Nations advocates have challenged the inappropriateness of these approaches given their lack of contextual and cultural acceptability [9,10,11]. Current HPE approaches that contribute to health professional reluctance to practice in rural communities and with marginalized populations are inappropriate and unacceptable for addressing the rural health inequities and workforce shortages of contemporary Australia.

### 1.2. Current Educational Approaches to Address Rural Health Workforce Shortages

Globally, educational efforts have been made to reframe health professional education to address rural health workforce shortages. Significant investments have been directed toward the design and delivery of longitudinal integrated placements/clerkships (LIPs/LICs) in the medical profession in response to prolonged rural generalist and general practice/physician shortages [12,13,14,15]. More recently, LIPs have been adapted to enable the design of similar immersive learning experiences for nursing [16]. In addition, there have been recommendations for universities to review their curricula and placement requirements to enable longer duration rural placements for allied health disciplines in Australia [17]. These models are currently anomalies in health professional education with short duration block placements across multiple settings continuing to be the standardized approach to education and curriculum design. As such, the evidence presented is heavily informed by medical student, academic, and clinical educator perspectives.

LIPs are defined by three core characteristics: (1) medical student participation in the provision of comprehensive care of service recipients over time; (2) the establishment and maintenance of learning relationships with clinicians; and (3) a student’s ability to achieve the majority of their core clinical competencies across multiple disciplines simultaneously [12]. While the positive educational impacts for medical students participating in LIPs are described in the literature, including the development of professional identity, improved patient-centeredness, higher performance on assessment tasks in comparison to peers, continuity of relationships, and deeper understanding of healthcare complexity in rural contexts [18], significant educational shortcomings have been identified. These include gaps between the planned and experienced curriculum [12], a lack of congruence between the rural context, curriculum, and assessment [18], student disengagement from learning opportunities not perceived as being of value to their core curriculum and assessment preparation [12], and the continuing dominance of biomedical interpretations of health that diminish the human experience [13].

It has been suggested that the educational and learning gaps experienced by students could be attributed to the lack of clinical teacher familiarity with the student’s core curriculum and learning needs, clinician work patterns, and work pressures [12]. The need to re-orientate medical curriculum beyond the biomedical model towards a curriculum ‘that does not reduce to hard science the rich complexities and ambiguities that being a human entails, but foregrounds human experience as integrous to the project of learning’ has also been acknowledged [13] (p. 1). Bartlett and colleagues (2020) describe an educational environment where assessment drives learning, necessitating a congruence between assessment tasks and curricular programs to ensure graduates meet the knowledge, skills, and attitudinal attributes required to fulfill accreditation and registration requirements [18]. Whilst the creation of a bespoke assessment process for students undertaking rural LICs may appear contextually relevant and responsive, it has been proposed that this tailoring would ‘open up the LIC, its students and teachers, to criticism of unequal, and potentially lower, outcomes and performance’ [18] (p. 13).

In contrast, the World Health Organization has recommended the revision of undergraduate and postgraduate curricula to ensure rural health topics and health professional education address the following: the needs of rural and remote populations; the health professionals undertaking their placement and practicing in these regions; and the different roles and scopes of practice required in these contexts [1]. The health workforce literature ‘largely reports delivery of standardized curriculum in rural places, with no explicit indication as to how rural concepts are being taught or constructed by students as a result of curriculum content, pedagogy, and learning assessment…Therefore, their teaching seems to come predominately from the context rather than from educational factors’ [2] (p. 30). The detrimental educational, learning and practice implications that result from this allegiance to the standardization of health professional curriculum is evidenced in the challenges confronted by the University Departments of Rural Health (UDRHs) in the ruralization and contextualization of health professional education in Australia.

### 1.3. University Departments of Rural Health

The Australian Government has made substantial investments in the Rural Health Multidisciplinary Training (RHMT) Program to address rural health workforce shortages [19]. Challenges exist beyond program parameters and the influence of RHMT funding on the UDRHs in achieving impactful rural health workforce outcomes. Comprehensive system-wide education, training, accreditation, registration, policy, and practice reforms are all needed to develop health professionals with the capacity to engage effectively in and across the interconnected systems that impact rural health, healthcare access, and outcomes [20]. Changes must include the transformation of HPE through a national policy focused on rural health equity and paramount consideration for the ruralization of HPE to influence rural practice uptake, career longevity in these contexts, and the health outcomes of rural and First Nations Peoples.

The UDRHs are well positioned to contribute to the development of health equity policies and to lead HPE reforms. The allocation of RHMT funding to nineteen Australian universities to deliver on rural health and workforce outcomes, the embeddedness of UDRH academics, the geographical distribution of these academics across all States and the Northern Territory, as well as their established university and community relationships, diverse practice backgrounds, and deep understanding of rural contexts elevate their collective capacity to contest the current status quo [21]. These rurally -based academics can authentically challenge urbanized and colonized health policy and HPE that contribute to the inadequate interpretation and misrepresentation of rural contexts and the deficit discourses drawn on in characterizing rural people, places, and healthcare delivery [22].

In response to the protracted nature of the health inequities and workforce shortages experienced, the Broken Hill UDRH invested in the development and implementation of the Anabranch Framework (AF) for the Ruralization of HPE. The AF systematically organizes inter-linked domains, constructs, and structural elements to provide a high-level strategy to reframe how rural health is theorized, taught, practiced, and integrated across Australian education, health, and community contexts. The AF is focused on the development of equity-orientated health professionals through tailored and place-based approaches to HPE, professional practice, and service provision. This paper describes the following: the longstanding barriers confronted by the Broken Hill UDRH in the ruralization of HPE; the evolution of the Broken Hill UDRH HPE contributions; the development, content, and utilization of the AF; the policy, registration, accreditation, education, and practice reforms needed to enable AF adaptation, implementation, and scalability; and a proposed approach to evaluating AF impacts and outcomes.

### 1.4. The Longstanding Barriers Confronted in the Ruralization of Health Professional Education

The Broken Hill UDRH has experienced multiple barriers in the ruralization of HPE. These barriers are situated in health workforce policy, curricula content, pedagogical approaches, placement and practice models, accreditation and registration requirements, and rural health systems (Table 1). Collectively, these barriers undermine the contextualization of HPE and the ability of the UDRHs to deliver on RHMT program parameters, rural health workforce outcomes, and ultimately, improvements in health equity attainment for rural populations.

### 1.5. The Evolution of the Broken Hill University Department of Rural Health’s Contributions to Health Professional Education and Service Provision

The Broken Hill UDRH was established in 1996/97 in far west New South Wales, Australia, to deliver on RHMT Program parameters including the provision of effective rural training experiences for health students and the development of an evidence base for the efficacy of training strategies in delivering rural health workforce outcomes [19]. The work of the Department has been underpinned by longstanding collaborations with multiple Australian Universities, health and school districts, Aboriginal Community-Controlled Health Organizations, non-governmental organizations, and private practices. This work has focused on the design, implementation, and evaluation of alternative strategies to support community-engaged and contextualized HPE and practice. This is evidenced by the UDRHs’ contributions to the development of innovative primary healthcare and community-engaged service-learning models for multidisciplinary health students and the redesign of primary healthcare services and practice [23,24,25,26]. Recently, the UDRH has focused on the development of evidence-informed longitudinal integrated placements (LIPs) for nursing and allied health students, reflecting the educational investments already afforded in medical workforce education [16,17]. These placement models are underpinned by the delivery of contextualized rural curriculum across extended placement durations and the extension of this curriculum into new graduate programs.

## 2. Methods

### 2.1. The Context

Compared with people living in metropolitan areas, Australians living in rural and remote locations have shorter lives, higher levels of disease and injury, and poorer access to and use of healthcare services [3]. In far west New South Wales, residents have a shorter life expectancy, are 2.5 times more likely to die from avoidable deaths, and twice as likely to die by suicide than their metropolitan counterparts [27]. McBride (2023) stated that ‘the far west is sick and not getting the medical attention it requires—particularly when compared to their city counterparts … alarm bells should be ringing for the far west’ [27] (p. 11). In parallel, the region also experiences significant health workforce shortfalls across all disciplines, undermining the capacity of health and related systems to address existing disparities. Given the gravity of this situation, there was an imperative to engage in alternative solutions to elevate healthcare access, health, and health workforce outcomes. This included action towards the ruralization of HPE in response to the ringing of these alarm bells.

### 2.2. Methods

**Figure 1:**
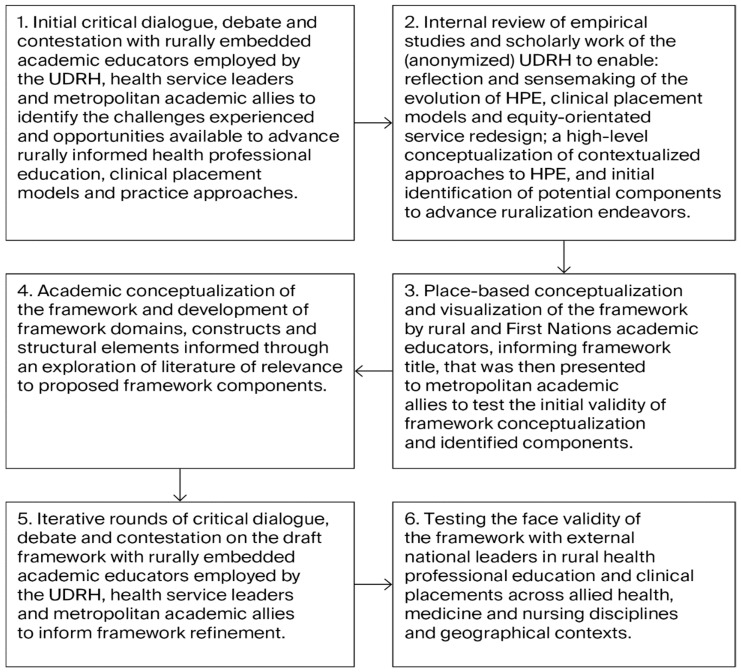
Process used to develop the Anabranch Framework. Adapted from McCormack B and McCance T [28].

The process of developing the Anabranch Framework was iterative, involving a series of systematic steps (Figure 1).

This process was informed by the work of McCormack and McCance (2006) in their development of a conceptual framework to inform person-centered nursing [28]. The methods used included initial critical dialogue, debate, and contestation with academic educators employed by the UDRH, rural health service leaders, and metropolitan academic allies to identify the challenges experienced and opportunities available to advance rurally informed HPE, clinical placement models, and practice approaches. The challenges identified have been described in Table 1. The identified opportunities to advance the contextualization of HPE, clinical placement models, and equity-orientated health care provision were drawn on to inform framework construction. An internal review and reflection on the empirical studies and scholarly work of the Broken Hill UDRH generated between 1996 and 2025 (n = 59 publications), focused on health professional education, clinical placements, and equity-orientated service re-design, was undertaken to ensure the centrality of place-based and contextualized evidence and approaches in framework construction (Table S1).

**Figure 2:**
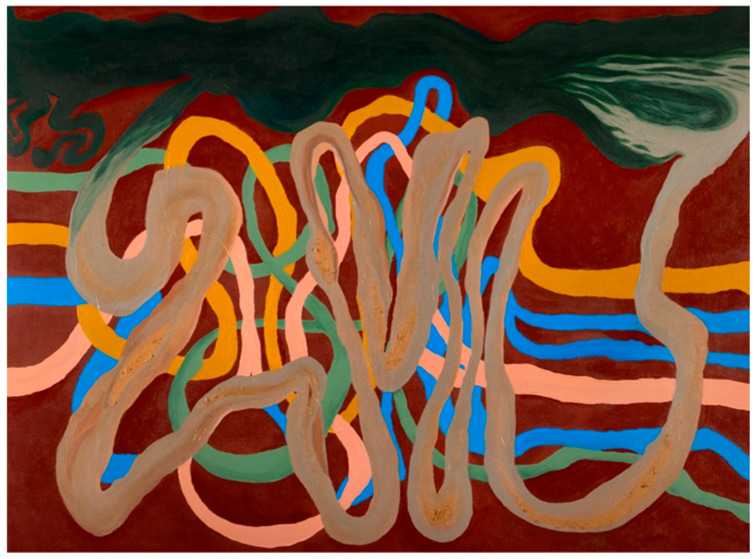
Place-based conceptualization of the Anabranch Framework.

This review included an internal reflection and sensemaking by rural academic educators to enable the synthesis of knowledge generated over time, nearly three decades, and disciplines, including allied health, medicine, and nursing. This initial process focused on a high-level conceptualization of the main ideas, relationships, and evolution of understanding, and the initial identification of potential components required to advance ruralization endeavors. Rural and First Nations academic educators then contributed to a place-based conceptualization and visualization of the framework. A visual image of the place-based conceptualization of the framework was placed on a whiteboard in an accessible area of the Broken Hill UDRH to enable critical dialogue, debate, contestation, and refinement of this conceptualization (Figure 2).

The place-based conceptualization grounds the framework in the landscape in which it has been developed, providing contextual relevance, applicability, and acceptability to and for rural and First Nations people and places, informing the frameworks title, the Anabranch Framework. An anabranch is a channel of water that leaves a major river system and then rejoins the river further downstream. In far west New South Wales, the Great Darling Anabranch mirrors the flow of the Lower Darling River before converging with the Murray River in southwest New South Wales [29]. The background colors of the place-based conceptualization reflect AF domains, constructs, and structural elements. The overlaying anabranch represents the complex and convoluted journey taken by health professionals as they progress towards the ruralization of their professions and practice. Once these health professionals have traversed through the anabranch, they rightly take their place alongside their professional peers who have remained within the originating HPE pathway. This reconvergence acknowledges the positionality of rural health professionals as an essential component of the nation’s health workforce. These health professionals will be noticeably different to their counterparts because of their alternative education, practice, and community experiences, reflecting the visible difference that exists when the clay-colored waters of the Darling Anabranch converge with the greener waters of the Murray River.

Rural and First Nations academic educators drew on the place-based conceptualization of the framework to interpret and translate this conceptualization through an academic lens, defining framework domains, constructs, and structural elements that were further refined through an exploration of the literature of relevance to the proposed framework components. Additional iterative rounds of critical dialogue, debate, and contestation on the draft academic framework were then undertaken with rurally embedded academic educators, health service leaders, and metropolitan academic allies to inform academic framework refinement. Testing of the face validity of the final draft of the framework was then undertaken with external national leaders in rural health professional education and clinical placements across allied health, medicine, and nursing disciplines situated in other rural Australian contexts. Of significance during this step was the expressed resonance of framework rationale and intent, its relevance across disciplines, and the ease with which these senior leaders were able to contextualize framework domains, constructs, and structural elements, signaling the relevance of the framework beyond the rural location in which it had been constructed.

**Figure 3:**
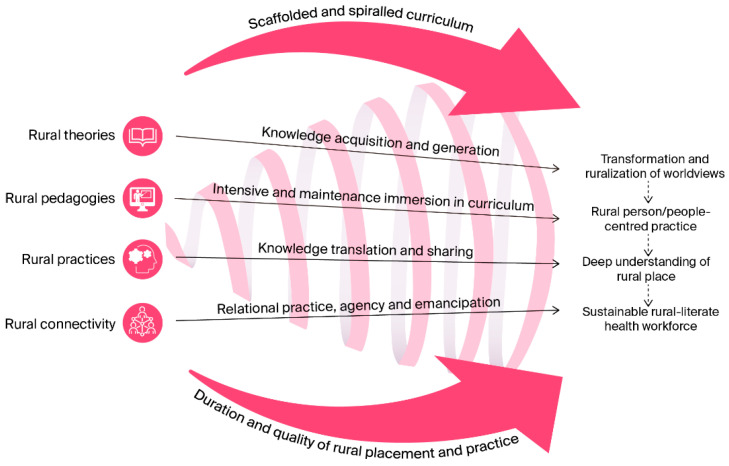
Academic conceptualization of the Anabranch Framework.

The development of the AF reflects a process grounded in long-term programmatic scholarship, critical dialogue, and reflective synthesis. Key informants involved in the conceptualization and design of the framework were knowledge contributors engaged in the process through their role-based expertise, long-standing practice relationships and engagement with rural health professional education and practice systems, reflecting interpretive and practice-informed traditions. The Anabranch Framework has been intentionally designed as a meta-level, integrative framework rather than a theory-specific model. Its function is to provide a contextual architecture within which multiple theories may be mobilized, adapted, or operationalized in rural, remote, and First Nations contexts. The final academic conceptualization of the AF (Figure 3), as well as framework domains, constructs, structural elements, and aspirational outcomes are now described.

## 3. Results

### 3.1. The Anabranch Framework for the Ruralization of Health Professional Education

The AF comprises four inter-related rural domains: theories, pedagogies, practices, and connectivity; four constructs: knowledge acquisition and generation, immersion in rural curriculum, knowledge translation, and sharing and relational practice; and two structural elements: spiraled and scaffolded curriculum and duration and the quality of rural placement and practice. Collectively, these domains, constructs, and structural elements contribute to four ambitious outcomes: the transformation and ruralization of health professional worldviews, the advancement of rural person-centered practice, the acquisition of a deep understanding of rural places, and the development of a sustainable rural-literate health workforce.

The following discussion focuses on AF components and their interconnectedness. The current utilization of the AF and the implications for policy, registration, accreditation, education, and practice reforms are described. Contextualized examples of AF domains, constructs, and structural elements are provided at the end of this section to guide framework interpretation and model design. Steps to inform the utilization and transferability of the framework are also provided. The authors caution that these contextualized components cannot be directly replicated in other rural locations given the need for tailored approaches, inclusive of local and First Nations protocols, to ensure AF responsiveness, relevance, and impact. The AF is a high-level strategy to guide the development of contextualized, place-based, and responsive HPE models.

### 3.2. Domain 1: Rural Theories

A theory is a set of inter-related constructs and propositions that presents a systematic view of a phenomenon, specifying relations among variables and with the purpose of explaining and predicting the phenomenon [30]. Theories provide conceptual frameworks that explain complex scenarios which help us ‘to better understand them, even to perceive them in different ways’ [31] (p. 341). In this instance, the AF seeks to provide an alternative perspective of the role of HPE in Australia in addressing rural health inequities and health workforce shortages and to challenge health professionals to reflect on their current framing and interpretation of rural healthcare and practice. Urbanized and colonized worldviews can determine the theories used to frame an inquiry into the complex phenomena of rural healthcare, resulting in the further marginalization of these populations, the amplification of harmful stereotypes, and the sharing of disassociated perspectives that can act to deter rural practice uptake [2,5,11]. The AF is underpinned by theoretical propositions that seek to advance a deeper understanding of rural health and the complex interplays that exist between HPE, health equity attainment, health workforce outcomes, service provision, and professional practice in rural contexts [32,33].

To achieve this, health professionals need to be exposed to multiple theories and experiences to support their sense making of rural health. It is ‘only by grasping the many different ways of seeing available to us that we can then understand the interchangeability of the lenses in our glasses and understand all the ways of seeing available to us’ [31] (p. 343). This necessitates the identification and delivery of theories that can be ruralized and theories that emerge in specific rural contexts, with specific rural populations. Careful consideration needs to be afforded to the theories selected in the utilization of the AF to ensure tailored approaches across Australia’s diverse rural contexts, acknowledging that theories do not ‘offer us insights into absolute truths; they offer explanations and detailed premises that we can wrestle with, agree with, disagree with, reject, and/or accept’ [31] (p. 343). The authors propose that a failure to identify and enact theories of real relevance to real rural people, places, and healthcare provision is at best naïve and at worst a contributing factor to the further entrenchment of paternalistic rural health policy, HPE, health service provision, and professional practice.

#### Construct 1: Knowledge Acquisition and Generation

Theories play a critical role in health professionals’ acquisition of the alternative perspectives and new knowledge required to tailor their practices to the needs of rural populations. Health professionals undertaking their placement and practicing in rural locations need support to generate new interpretations and perspectives that enable them to challenge systems, policies, processes, and structures that contribute to health inequities and health workforce shortages. The AF specifically focuses on disrupting traditional knowledge hierarchies and expertise in HPE, those that privilege scientific, urban, and colonizing knowledges over local knowledges and lived experiences. This disruption seeks to highlight the disconnect that can exist between policy makers, educators, and practitioners and the knowledge and expertise held in rural communities and populations, elevating insights into the capacity rural populations have to draw on internal attributes to drive the changes required to improve their own health outcomes [22,33]. The legitimization of rural and First Nations knowledges provides alternative insights into how knowledge is acquired and from whom, promoting a more democratized understanding of rural health, people, and places. As stated by Lowe et al., expertise is distributed and ‘embraces the full and rich diversity of types of human knowledge, experience, and skills, whether tacit or codified, informal, or certified, individualized or collective’ [34] (p. 29).

### 3.3. Domain 2: Rural Pedagogies

In achieving this level of knowledge democratization in HPE and practice, transformational emancipatory pedagogies (TEPs) are required. TEPs challenge educators to move beyond competency-driven learning to produce empowered graduates who understand the importance of ‘amplifying marginalized voices, advocating for education that challenges marginalization and exclusion and promotes social justice and transformative change’ [35] (p. 44). TEP is synergistic with rural HPE and practice given the deficit discourses used, the imbalance of power and privilege that can exist between universities, health systems, and the rural communities they serve, and the role health professionals can play in addressing the confronting inequities and social injustices that exist.

TEPs position educators and health professionals as co-creators of knowledge, further extending the democratized approaches required to transform rural healthcare and practice. TEPs are focused on ‘individual emancipatory goals, such as nurturing critical consciousness about one’s situation in the world, and collective emancipatory goals, such as a greater awareness of social injustice and a desire to seek socio-political transformation’ [36] (p. 8). The TEP drawn on in the construction of the AF accounts for the disorientating and ethical dilemmas that can be confronted by health professionals, specifically when their pre-existing perceptions of rural healthcare are disrupted, new ways of interpreting the causalities of inequity are needed and alternative solutions are required [37]. These TEP approaches are intrinsically linked to the AF structural element of spiraled and scaffolded curriculum.

#### Construct 2: Intensive and Maintenance Immersion in Curriculum

To support the level of transformation required, there is a need to engage health students and professionals in intensive immersion in rural curriculum in response to the existing theoretical, knowledge, and practice deficits [2]. Intensive immersion focuses on preparing health professionals for the real-world complexities associated with rural healthcare delivery and alternative healthcare practices. Maintenance immersion is needed to elevate health professionals’ awareness of social injustices, to provide them with opportunities to engage in actions that seek to transform socio-political and health structures that marginalize rural and First Nations Peoples, and to mitigate their re-urbanization and re-colonization. These immersive approaches support health professionals to transition from superficial interpretations to deeper levels of knowledge acquisition, enabling them to contextualize and integrate their abstract theoretical knowledge of rural health with authentic and culturally respectful ways of knowing, being, and doing healthcare in rural contexts [38]. Immersive curriculum approaches are aligned with the AF structural element of duration and the quality of rural placement and practice.

### 3.4. Domain 3: Rural Practice

Rural practice differs significantly from healthcare provision in urban health systems, reinforcing the need for rural theories, pedagogies, and authentic exposure to the alternative health service and practice approaches evidenced in these contexts [2]. Geographical isolation, population demographics, political and policy imperatives, the limited representation of health professionals, and specialist services necessitate greater autonomy in practice and healthcare integration to ensure the needs of rural populations are addressed [2,39]. The AF has been designed to enable these alternative experiences and to support health professionals in transforming their practice to align with public health and primary healthcare models.

The Australian Government’s Final Report from the Unleashing the Potential of our Health Workforce—Scope of Practice Review (2024) identified that ‘rural and remote practice requires the development of a range of skills and capabilities not routinely taught outside this setting’ [39] (p. 98). A broader scope of practice for rural health professionals is necessary in responding to the complex health needs of rural populations and communities. A range of acute, community organizations, primary healthcare organizations, Aboriginal Community-Controlled Health Organizations, private practices, and non-government organizations are present in rural locations, providing tailored health programs and practice approaches. The diverse nature of these organizations in rural and remote contexts provides quality training grounds for health professionals [39]. Training opportunities cannot be delivered in isolation from HPE that promotes a deeper understanding of the unique context in which healthcare is delivered and the health needs of rural people. The AF aligns with the Australian Government’s proposition that ‘addressing the challenges that face the development of the rural/remote primary health workforce requires a broad national reform agenda that dovetails with local community-based needs’ [39] (p. 101). This national reform agenda must encompass HPE to ensure the development of a dynamic and equity-orientated rural health workforce.

#### Construct 3: Knowledge Translation and Sharing

The AF connects rural theory and pedagogy to practice, enabling knowledge translation in real healthcare and community settings. The Canadian Institutes of Health Research (2018) defined knowledge translation as ‘a dynamic and iterative process that includes synthesis, dissemination, exchange, and ethically-sound application of knowledge to improve health, provide more effective health services and products, and strengthen the healthcare system’ [40]. In advancing the democratization of knowledge acquisition and generation, alternative approaches are required to support health professionals to translate local knowledge into their practices and in sharing the knowledge acquired with their broader communities of practice (CoPs) [41]. CoPs are drawn on in rural health settings to support knowledge translation, implementation, and sharing within and between individuals and the organizations in which placements are undertaken and health professionals practice.

CoPs are defined as ‘groups of people who share a concern, a set of problems, or a passion about a topic and who deepen their knowledge and expertise in this area by interacting on an ongoing basis’ [41] (p. 2). As knowledge sharing in CoPs increases in meaning and use, the generation of new knowledge is promoted. However, more is needed to address rural health inequities and health workforce complexities; knowledge sharing needs to translate into impactful action towards health equity attainment [41]. The AF is focused on developing health professional CoPs that advance the skills, knowledge, and capabilities of these professionals to act independently and collectively towards actions that promote health equity and social justice attainment, with and for rural populations.

### 3.5. Domain 4: Rural Connectivity

CoP also contribute to the creation of a stronger sense of connection to rural people and places, a key factor in influencing rural career preference and uptake. The greater the immersion of health professionals with CoPs in rural contexts the greater the capacity they have to develop connections through repeated interactions with their healthcare teams and the rural populations they serve. Connectivity ‘is dynamic, occurring in overlapping academic, social, personal, and physical spaces’ and extends beyond ‘feeling connected to peers, to also include staff and the wider learning environment’ [42] (p. 194). Positive connections between clinical, educational, and social environments, and the relationships formed with peers, healthcare teams, communities, and multidimensional CoPs can promote health students’ and professionals’ sense of being valued, trusted, and belonging in rural contexts. Jones et al. (2025) also describe the importance of extending this level of connectivity to include the establishment of deeper connections, those required to bridge the divergent worldviews that can exist between higher education, health systems and professionals, and rural populations [16]. The AF seeks to elevate health professional connectivity in rural contexts as a pathway to the enactment of relational practice, agency, and emancipation.

#### Construct 4: Relational Practice, Agency, and Emancipation

Relational practice and agency are precursors for the advancement of rural health professional and community emancipation from the systems, structures, policies, and practices that act to marginalize them. Relational practice is a transformative approach to health professional practice, acknowledging the increasing complexities confronted in healthcare delivery, the impact of entrenched inequities on health outcomes, and the risks associated with the de-contextualization and depersonalization of healthcare for rural populations. Relational practice calls on health professionals to look ‘beyond the surface of one-to-one encounters, by considering what shapes those encounters’, enabling them to direct their actions ‘in ways that foster trust, respect, compassion, and mutuality’ in their caring relationships [43] (p. 203).

Trust is critically important in the provision of rural healthcare and is considered an attribute of rural community-literate practice [5]. Within primary healthcare, longitudinal contact is one part of relational continuity that contributes to a trusting relationship, the establishment of a shared history between service recipients and providers, and the creation of accountability and mutual commitment [44]. Relational practice is a precursor to the establishment of relational agency, the ability of health professionals to ‘recognize the social and material nature of individuals’ existence, and the crucial role that structural factors have in shaping their ways of being, thinking, and acting’ [45] (p. 2). Relational agency encompasses the ability of individuals to resist, destabilize, transform, and reconstruct the discourses that act to define them. It is through the establishment of relational practice and agency that health professionals can contribute to the emancipation of rural communities and populations from inequitable policies, practices, structures, and processes. Emancipation is a form of ‘liberation from the unjust relationships that bind individuals with the projective intention to bring about a future which is different from the present and the past’ [45] (p. 2). Relational practice, advocacy, and emancipation are of importance in the AF for the development of rural health professionals who have the capacity to embody morally authentic ways of practicing through the creation of health services that align with their own values and the values and needs of the rural populations they serve.

### 3.6. Structural Elements

#### 3.6.1. Spiraled and Scaffolded Curriculum

Spiral curriculum is situated in constructivism where learners actively contribute to knowledge acquisition and translation by building on the concepts they are exposed to in a cyclical manner, reflected in the helix depicted in the academic conceptualization of the AF. The key stages associated with a spiral curriculum are the logical sequencing of topics, reflecting on concepts delivered throughout a program to enable learners to transition from simple to complex interpretations through higher-order thinking and the integration of new learning with previous learning [40]. Spiral curriculum is ‘a compelling approach in higher education for fostering deep learning and competency development’ [46] (p. 25). The inclusion and implementation of spiral curriculum in the AF has been informed by the work undertaken by academics embedded in the Broken Hill UDRH to advance the design and delivery of ruralized curriculum for final year health students participating in LIPs [16,17]. These contributions highlighted the importance of rural academics investing time towards comprehensively understanding the existing curriculum, competency, and knowledge requirements to enable the integration of the ruralized curriculum, alignment of the ruralized curriculum to core learning outcomes, and the elevation of student capacity to achieve higher-order conceptualizations of rural healthcare and professional practice. These processes also sought to minimize student cognitive overload and informed the inclusion of structured and opportunistic critical reflection sessions to support student engagement with their core curriculum, the rural curriculum, and their practice experiences.

Furthermore, the mapping of existing curriculum with the ruralized curriculum enabled knowledge scaffolding that accounted for the introduction of new theoretical propositions and practice approaches. Van De Pol et al. (2010) defined scaffolding as ‘temporary support provided by an educator to aide students in completing a learning task that would prove difficult without such support’ [47] (p. 272). The scaffolding of the ruralized curriculum across placement durations elevated students’ capacity to adapt to new social and clinical contexts and the complexities and demands confronted during their extended placement experience. The importance of contextualizing, deconstructing, and co-constructing theoretical knowledge through the integration of rural perspectives was found to be critical to effective learning. This approach to scaffolding reinforces student-centered learning in navigating rural healthcare environments, healthcare complexity, and evolving healthcare systems and communities [47].

#### 3.6.2. Duration and Quality of Rural Placement and Practice

The utilization and impact of the AF is heavily dependent on the duration and quality of rural placements and practice. Extended timeframes are required to enact AF domains, constructs, and structural elements. This includes the need for the extension and integration of LIPs across disciplines to promote connectivity between health students and service recipients, supervisors, and communities. This level of connectivity is fundamental to student learning and an important component in the preparation of health professionals for future rural practice [48].

Whilst medical education has embedded rural LIPs into the curriculum, nursing and allied health disciplines have not yet been afforded these same immersive learning opportunities [16,17]. Placement durations for these disciplines are typically short and necessitate multiple placements, across multiple settings, and with different caseload types to meet competency requirements. These placement models undermine place-based endeavors to integrate a ruralized curriculum into placement and practice experiences. Where efforts are made, they can be perceived as placing additional learning burdens on students, lacking relevance and alignment to core assessment requirements, and detracting from student contact with service recipients to gain the clinical competencies required. Furthermore, universities can be reluctant to engage in rural LIP innovations given the implications for curriculum content, placement structures, and processes [18]. The direct translation of a metropolitan curriculum into the existing and emerging end-to-end and LIP programs in rural Australia is of grave concern given the lack of program tailoring to rural contexts [2].

Challenges in the retention of a rural workforce further constrain the delivery of HPE and the transformation of health professional education and practice. The frequent turnover of staff at the executive, management, and direct service provision levels has detrimental consequences for driving the strategic changes required for the ruralization of HPE. Staff attrition disrupts university–health systems relationships and diminishes professional opportunities to engage with communities and in alternative interpretations of healthcare provision. The reliance on high-cost and transient agency staff also creates resource constraints that detrimentally impact resource allocations towards ruralization strategies.

The authors propose that this current environment perpetuates a lack of quality placement and practice experience for health students, professionals, health systems, and the communities they serve. In contrast, the AF seeks to promote quality learning experiences through the design of multidisciplinary LIPs underpinned by a ruralized curriculum, the establishment of multidisciplinary CoPs, the integration of the curriculum into health professional career pathways and in the health service sites in which placements are undertaken, and professional practices to mitigate the challenges confronted.

As previously described, the AF is a high-level strategy to inform the collaborative design of rural place-based models of HPE. The authors propose that the AF can be utilized to inform the development of indigenized HPE models (i.e., the indigenization of AF domains, constructs, structural elements, and outcomes). This would necessitate comprehensive and culturally respectful engagement and consultation with the leadership of First Nations Peoples, academics, and health professionals in consideration of the cultural acceptability of the AF and the advancement of culturally responsive Indigenized models. The AF is not prescriptive and has been constructed with the intent for adaptation and contextualized interpretation and utilization. To further support the enactment of the AF, the authors have provided contextualized examples of the AF in action in one rural context (Table 2). The AF’s domains and constructs have been integrated into this table to highlight the complex interplay that exists between these components.

### 3.7. Framework Outcomes

The outcomes of the AF are aspirational, reflecting the results expected from the ruralization of HPE. These outcomes are the transformation and ruralization of health professional worldviews, the advancement of rural person-centered practices, health professionals’ acquisition of a deeper understanding of rural places and the development of a sustainable rural-literate health workforce—a workforce equipped with the knowledge, skills, and attributes required [2] to address the health inequities experienced by rural and First Nations Peoples.

## 4. Discussion

### 4.1. Framework Utilization

The AF is being used by Broken Hill UDRH academics and researchers to guide the changes required to implement and evaluate the ruralization of HPE in far west New South Wales. These academics are transitioning from training strategies focused on delivering metropolitan curricula to health students undertaking their placement in the region to the ruralization of HPE theories, pedagogies, and practices that are of relevance to rural and First Nations Peoples; working as a transdisciplinary team leading the design and delivery of the ruralized curriculum; and aligning their educational contributions towards health equity-orientated practice.

To enable this transition, the Broken Hill UDRH has focused on the development of LIPs for nursing and allied health disciplines to align with the existing medical student placement durations. The alignment of longer placement durations has facilitated a new transdisciplinary approach to HPE and the collective engagement of rural academics in the construction and implementation of the AF. These academics are now actively reflecting core components of the AF in their own educational practices through the following: (1) the acquisition of new peer knowledge, relational practice, and the collective generation of new approaches to HPE; (2) embedding intensive and maintenance immersion in rural curricula across disciplines through aligned orientation, education, critical reflection sessions, and case studies; (3) the contextualization of practice experiences; and (4) the translation of these new approaches into their educational practice and curricula delivery. This new body of work is now being extended to support new graduate nurses transitioning to practice in the region.

### 4.2. Implications for the Utilization of the Anabranch Framework

The authors acknowledge the policy, accreditation, registration, education, and practice implications associated with enacting the AF. The authors also acknowledge that the UDRHs lack the positionality and authority required to drive the multi-system changes required. Currently, the Broken Hill UDRH is dependent on soft power to attract and persuade universities to engage in alternative approaches to HPE and their collaborative capacity to work with regional health services to advance these strategies. Challenges exist in the effectiveness of soft power in influencing policy makers and universities who may have an aversion to the level of disruption required to ruralize HPE and to take social responsibility for the health workforce shortages, health inequities, and social injustices confronting rural and First Nations populations.

If the UDRHs are to be more impactful, then the leaders of these Departments and their universities must take collective action towards rural health equity and the ruralization of HPE. As stated by Kemple (2019), we need leaders who are ‘assertive, have command of high-quality information, and understand policy processes, competing influences, and context for the policy maker but who are flexible in their approach and can build the constructive “honest broker” relationships that result in the best outcomes’ [49] (p. 1). Table 3 describes the strategies required in Australia to advance the AF and the ruralization of HPE and situates these with relevant government, higher education, accreditation and registration, and health systems domains.

### 4.3. Transferability of the Anabranch Framework

The authors acknowledge the complexities that may be experienced in the adoption, translation, and scalability of the framework across diverse geographical, educational, health service, and community contexts. Table 4 provides guidance on recommended steps to support framework transferability, acknowledging the sensitivity required to ensure local governance, autonomy, and protocols are respected. The order of these steps is inter-changeable, and they should be enacted in response to the discrete context, policy, strategic, educational, and relational environments that exist.

The authors propose that the following minimal criteria are required to enable the transferability of the AF: the presence of rurally embedded health academics with close linkages to regional health and education stakeholders and metropolitan academic allies; access to educational infrastructure (i.e., safe learning environments, teaching spaces, physical or IT-enabled spaces for educational delivery and critical reflection); access to rural health and curricula expertise to inform the design, implementation, and evaluation of HPE models underpinned by the AF; sustainable funding to support academic and key stakeholder contributions to the adaptation, implementation, and evaluation of the AF and associated models; and policy maker, academic, and place-based willingness to engage in HPE innovations. In the Australian context, the UDRHs have the capacity to address the minimal criteria described through policy mandates, program parameters, Commonwealth funding, university and community connectivity, and they have the capacity for place-based responsiveness.

### 4.4. Evaluating the Anabranch Framework

Historically, HPE has applied reductionist evaluation approaches that focus on a linear attribution of relationships between specific actions and outcomes [51]. Increasingly, these approaches are considered ‘incompatible with the complexity of HPE where contextual factors dynamically interact and exert variable influence on outcomes’ [51] (p. 3). The AF has been designed, and is being implemented, in an environment where internal and external political, policy, education, health, funding, and contextual factors interact, contributing to high levels of uncertainty and ambiguity. These circumstances directly and indirectly influence HPE innovations in rural contexts, their impacts, and outcomes [52]. Hence, the development of the AF research and evaluation protocol is being guided by complexity theory [52,53] and the CIPP (Context, Input, Process, Product) program evaluation model [54,55] to provide greater insight into how these factors influence AF design, implementation, impacts, and outcomes.

The four components of evaluation of the CIPP model will be used—context, input, process, and product evaluations. All are important for understanding what has worked, including where the program can be adapted and strengthened, and for assisting the assessment of program outcomes across participant groups and sub-groups to determine overall program impacts, outcomes, effectiveness, transportability, and sustainability [54,55]. The importance of program evaluation in understanding how programs work in practice is now recognized as being vital in building an evidence base that informs policy and practice. The Broken Hill UDRH has commenced working with a range of AF stakeholders in the development of this protocol and is in the process of re-distributing internal resources to enable access to the research expertise required to advance the four sets of evaluation studies that will be undertaken. In preliminary consultations with key stakeholders, the following examples of questions of relevance to each study have been proposed: Context study: How does Australia’s health workforce policy impact health professional student and professional capacity to engage in ruralization endeavors? Input study: What resources are required to support the conceptualization, design, implementation, evaluation, sustainability, and transportability of the AF? Process study: What changes and adaptations were required to create a transdisciplinary academic team to lead the delivery of the AF? Product Study: Did the AF achieve the aspirational outcomes proposed? What positive and negative outcomes, intended and unintended outcomes, and short-, medium-, and longer-term outcomes resulted from the utilization of the AF? Ongoing consultations will inform the identification of the most appropriate methods to answer these questions, the identification of relevant measurements to determine the effectiveness of the AF in meeting the aspirational outcomes described and AF impact on policy, education, practice, workforce, and health equity outcomes.

### 4.5. Limitations

The AF has been conceptualized and constructed in one specific geographical location in rural Australia. This may have implications for the transferability and scalability of the AF to other rural contexts where health and higher education systems engagement, community relationships, resourcing, service collaborations, and university commitments may be variable. The place-based conceptualization of the framework presented in this paper does not preclude others from reconceptualizing the framework to reflect the unique contexts that are represented across rural, remote, and First Nations communities and regions. Comprehensive research will be required to provide evidence on the acceptability, impacts, and outcomes of AF implementation on the ruralization of HPE, rural health workforce outcomes, and advancements towards health equity attainment. Two studies are currently underway to explore AF participation impacts for nursing and allied health students undertaking LIPs in the region. A third study is currently being developed to explore the impact of participation in a multidisciplinary LIP model underpinned by the AF. Research is also required to explore the political, policy, higher education, and health systems impacts associated with AF adoption, adaptation, implementation, and scalability.

## 5. Conclusions

The AF is centered on transforming HPE for health students, professionals, and the rural communities they undertake their placements and practice in as a foundational approach to addressing critical rural health workforce shortages and the preparation of health professionals in advancing rural health equity attainment. The AF has been specifically constructed to challenge and inform HPE in Australia. The authors propose that the AF is of international relevance for other rural and First Nations communities experiencing similar pervasive health inequities and health workforce shortages. The AF highlights the importance of theoretically informed HPE in rural contexts, tailored pedagogical approaches that disrupt traditional knowledge hierarchies and privilege, practice experiences that align curriculum and service provision to community needs and contexts, and the importance of connectivity between higher education, health systems, professionals, and the rural populations they serve.

While acknowledging the need for pragmatic, incremental, and sustained change towards the ruralization of HPE and practice in Australia is important, there is urgency for educational reforms to rapidly address the critical rural workforce shortages and entrenched inequities experienced. This will necessitate engagement with alternative evaluation approaches that move HPE insights beyond reductionist interpretations to those that seek to understand the complexity and programmatic implications and impacts of AF utilization in the ruralization of HPE. The priority needs to be the transformation and ruralization of health professional worldviews, the advancement of rural person-centered practices, health professionals’ acquisition of a deep understanding of rural places, and the development of a sustainable rural-literate health workforce. The AF provides a comprehensive approach towards disrupting the current status quo and towards the development of a more socially responsible, inclusive, rurally responsive, and equity-orientated rural health workforce.

## Figures and Tables

**Table 1:** Barriers confronted in the ruralization of health professional education and consequences.

Barriers	Consequences
Health workforce policy	
Lack of cohesive and integrated national rural health workforce policies and governance structures, including across professional disciplines.	Multiple, fragmented and poorly aligned policy directives with no overarching governance structures across Commonwealth, State, Territory and regional government levels and professional disciplines.
	Complexity in navigating diverse, and at times conflicting, policy directions and priorities.
Short-term solutions and reactive interventions	Failure to engage in long-term, sustainable and contextualized rural health and health workforce solutions.
Supply-focused strategies dependent on non-resident agency, fly-in-fly-out service providers.	High cost, silo approaches that fail to consider health workforce readiness for rural practice, recruitment, retention, employment and cross-sector solutions.
Health professional education	
Lack of rurally informed content in health professional curricula.	Dominance of metrocentric and colonizing curricula.
Lack of clarity on how rural theories are identified and utilized to inform health professional interpretations of rural practice.	Maintenance of deficit interpretations and misrepresentations of rural and First Nations health, people, places and practice.
Privileging of scientific knowledges over rural and First Nations community scholars lived experiences.	Devaluing and marginalization of rural and First Nations Peoples’ knowledges, experiences, perspectives and expertise in informing HPE and healthcare delivery.
Curricula designed to meet the planning needs of universities not rural populations.	Disconnect and tension between metropolitan curriculum structures and processes and the needs of rural populations.
Lack of opportunity to scaffold and spiral HPE across rural health career trajectories.	Superficial interpretations of rural contexts and care.
	Re-urbanization and re-colonization of health professionals and their practice.
Metrocentric HPE layered into rural contexts.	Failure to tailor HPE to rural contexts, health needs and practice approaches.
Pedagogical approaches	
Educators with limited to no authentic experience of rural and First Nations healthcare delivery.	Pedagogical practices that act to decontextualize and entrench deficit interpretations of rural health.
Pedagogical approaches informed by content saturation.	Lack of engagement with rural relevant transformational and emancipatory pedagogies.
HPE delivered predominately in, and based on, metropolitan contexts.	Inclusion of ruralized HPE viewed as curricula add on/burden.
Rural educators expected to deliver metrocentric curriculum for longer term student placements in rural contexts.	Failure to tailor HPE to contexts and a devaluing of rural knowledges and expertise in the delivery of HPE.
	Fragmentation of theory, pedagogy and practice in rural contexts.
Placement and practice models	
Competency driven practice experiences for HPE students.	Student ability to engage in person-centered care diminished.
Dominance of hospital and illness focused placements and healthcare delivery models.	Devaluing of public health and primary healthcare practice.
Fragmentation and separation of theory to rural practice experiences.	Metrocentric placement and practice models layered onto rural contexts reducing opportunities for authentic, contextualized and rich learning.
Focus on short duration placements across multiple settings and with multiple case types.	Host site fatigue, perceptions of student churn through rural communities.
	Limited opportunities to engage health professionals in rural education, practice, healthcare teams and communities.
Policy, education, health and practice systems, processes and structures undermine the establishment of deep connections with community.	Poor alignment of training strategies to the needs of rural people.
	Poor educational preparation to ensure successful rural placement and practice experiences.
	Limited health professional knowledge and skills to respond to disruptive and ethical dilemmas in rural contexts.
Limited priority placed on relational practice in healthcare delivery	Lack of continuity and consistency in healthcare relationships.
	Capacity to consolidate knowledge informed by respectful and trusting relationships is constrained.
Lack of capacity building towards community engaged models of care and practice.	Failure to appreciate community engaged approaches to healthcare delivery resulting in transaction engagement experiences.
	Further entrenchment of metrocentric and colonized approaches across career continuums.
	Lack of reflective practice to inform critical thinking in rural practice development and adaptation.
Diminished quality placement experiences that enable the acquisition of new knowledges and practice approaches and the consolidation of ruralized healthcare delivery.	Placement and practice durations fail to: reflect educational evidence, meet community expectations and advance endeavors to ruralize HPE.
Accreditation and registration requirements	
Lack of accreditation and registration requirements to drive the inclusion of rural theory, practice and assessment in HPE.	Rural health is devalued in higher education systems and not prioritized as a core requirement to meet assessment, accreditation and registration requirements by educators, health students and health professionals.
Misinterpretation and misrepresentation of accreditation and registration requirements to maintain current HPE status quos that meet university needs.	Constrained ability to drive rural HPE innovations.
	High dependence on relationships between UDRH and metropolitan academics to identify and engage universities with an appetite for ruralisation endeavors
Rural health system fragility	
Existing health workforce shortages and staff fatigue.	Limited capacity to place students.
	Staff focus on delivering core services (perceptions of supervision and education burdens)
Frequent leadership change across health sectors.	Disrupted relationships and strategic collaborations.
Scare resources to support substantive and sustainable health workforce im-pacts.	Under-resourced initiatives.
High dependency on agency and transient health professional workforce.	Scarce resources redirected to high-cost external service providers, amplifying relationship and service fragmentation.
Lack of financial and human resources to support new graduate employment pathways.	Dependence on attracting more senior/qualified clinicians resulting in unfilled vacancies, routine staff turnover, lack of or fragmented healthcare provision and limited investment in comprehensive rural-centered career pathways.

**Table 2:** Contextualized examples of Anabranch Framework domains, constructs, and structural elements.

Framework Components	Contextualized Approach
Domain 1: Rural theories	Universal Theories: Complexity and complex adaptive systems, Ecological Systems, Relational Practice, Knowledge Systems.
	Rural relevant theories: Geographical narcissism, Comprehensive Primary Healthcare, Community engagement, Local knowledges including First Nations knowledge systems, Community Literate Healthcare, Emancipatory practice in rural contexts.
Construct 1: Knowledge acquisition and generation	Disrupt traditional knowledge hierarchies that privilege scientific knowledges over rural and First Nations Peoples knowledges. Utilize engagement strategies that recognize and legitimize rural and First Nations Peoples knowledges.
	Challenge knowledge hierarchies towards knowledge democratization.
Domain 2: Rural pedagogies	Engage rural and First Nations community scholars in narratives that link health professional head (cognitive), hands (practical) and heart (emotional) domains with lived experience.
	Engage rural academics in the design, delivery and evaluation of AF utilisation
	Structured and opportunistic critical reflection sessions to address disruptive dilemmas.
Construct 2: Intensive and maintenance immersion in curriculum	Structured intensive rural curriculum delivered at commencement of placements and maintenance engagement in curriculum across placement duration for health students.
	Structured intensive curriculum delivered at commencement of new graduate practice or employment in regions and maintenance engagement in curriculum across employment duration for health professionals.
	Open access to ruralized HPE sessions delivered.
	Intentional integration of ruralized HPE into structured multidisciplinary and interprofessional training programs.
Domain 3: Rural practice	Focus on student exposure to a range of health services situated beyond traditional hospital settings.
	Engage health leaders and professionals in the design of alternative placement and practice models.
	UDRH support the preparation of host sites for placement experiences.
	Establish close collaborations with health professionals practicing in these contexts and health organizations.
	Reframe healthcare provision towards public health and primary healthcare.
Construct 3: Knowledge translation and sharing	Leverage the CoP that are embedded in the nature of the work undertaken by UDRH.
	Bring together students from multiple disciplines to establish CoP, including engagement with multiple universities to achieve this outcome if required.
	Engage health professionals from across multiple disciplines, services and settings, and the communities they serve with student CoP.
	Provide structured education opportunities for CoP.
	Encourage social integration opportunities for CoP.
	Reconvergence of ruralized health professionals with their mainstream peers to elevate opportunities to extend knowledge translation, sharing and health equity action across CoP beyond rural locations.
Domain 4: Rural connectivity	Education connectivity: connectivity created between theory, pedagogy, and practice.
	Social connectivity: Structured opportunities to connect health students, professionals and the communities they serve through social engagement in community events and organizations.
	Practice connectivity: Immersion learning aligned to theory and practice, engagement in CoP.
	Focus on transformation of worldviews.
Construct 4: Relational practice, advocacy and emancipation	Inclusion of respect, trust, mutual benefit and reciprocity theory and principles in ruralized curriculum.
	Include a focus on empathy, active listening, and collaborative partnerships in advancing health equity endeavors.
	Challenge socio-political structures that contribute to rural marginalization.
Structural element 1: Spiraled and scaffolded curriculum	Mapping of core curriculum to ruralized curriculum.
	Focus on ensuring assessments are contextualized and of relevance to rural populations and practice.
	Student and health professional centered approaches to support learning.
	Mitigate cognitive overload through scaffolded learning and critical reflection.
	Employ academics with the required skills to construct and deconstruct complex theories, contexts and practice experiences.
	Provide additional resources to promote learning in context.
Structural element 2: Duration and quality of rural placement and practice	Advance multidisciplinary longitudinal integrated placements in collaboration with universities and rural health organizations.
	Design models that link short placement experiences to consideration of uptake of longer duration placements providing a structure for multiple supported and connected rural placements.
	Work with health organizations to embed ruralized HPE into new graduate and new employee programs and processes.
	Engage new graduates and new employees in existing CoP.

**Table 3:** Strategies required to advance the Anabranch Framework for the ruralization of health professional education.

Domains	Strategies Required
Government	Development of a comprehensive National Skills and Capability Framework for rural practice that consolidates rural generalist allied health, medicine and nursing knowledge, skills, and competency expectations
	Extend the role of the Office of the Rural Health Commissioner to have carriage of reporting through to Health Ministers on the transferability, scalability and impact of AF utilisation.
	Develop policy that guides RHMT funded universities towards the implementation of rural longitudinal integrated placement opportunities for nursing and allied health disciplines that is aligned to UDRHs capacity to place students in these alternative models.
	Extend the contributions of Regional Training Hubs to include nursing and allied health rural generalist career pathways.
	Extend the role of UDRH in the ruralization of HPE across health professional career continuums and resource them appropriately to enable them to undertake this extended body of work.
	Engage with RHMT funded universities to advance the inclusion and integration of rural curriculum across health disciplines and at pre and post registration health degree levels.
	Ensure Commonwealth funded longitudinal integrated placements and end-to-end training programs delivered in rural contexts are ruralized and show evidence of rural curriculum design, educational delivery, practice experiences and assessments of relevance to rural people, places and practice.
	Extend the financial supports provided to nursing and social work students to undertake placements through the Commonwealth Prac Payments scheme to allied health and medicine disciplines.
	Provide additional financial incentives for students participating in rural longitudinal integrated placements and end-to-end training programs.
Higher education	Map existing health professional curricula to the AF to identify theoretical, pedagogical and practice alignment to rural healthcare provision.
	Implement an ongoing program of education, promotion and adoption of the AF to inform the design, delivery and monitoring of HPE ruralization.
	Include high-quality rural education and training experiences in professional entry and post graduate education programs that encompass rural theories, pedagogies, practices and assessment criteria.
	Collaborate with UDRH in the establishment of multidisciplinary rural longitudinal integrated placements and end-to-end training programs to ensure tailoring to rural contexts, health needs and professional practice.
	Upskill educators to ensure relevance and responsiveness of rural health curricula.
	Establish structures and processes that engage rural health academics, health organizations and community representatives in curricula redesign and implementation.
Accreditation and Registration authorities	National Boards and accreditation authorities regularly review the AF to align accreditation and registration functions relating to rural standards, codes, competencies and guidelines for nationally regulated health professions practicing in rural contexts.
	Professional bodies regularly review the AF to align accreditation and professional standards, functions relating to standards, codes, competencies and guidelines for self-regulated health professions
	National Boards and professional bodies elevate health professional education assessment, education and practice expectations to include ruralization strategies and interprofessional domains, including multidisciplinary longitudinal integrated placement experiences in rural contexts.
Health Systems	Establishment of university and rural health systems collaborations to advance HPE ruralization. Appointment of strategic rural workforce and education leads in State and Territory health sectors.
	Distribution of additional resources from Commonwealth and State Governments to rural health services to support the transition and implementation of HPE ruralization strategies across directorates and recruitment and retention initiatives.

**Table 4:** Guidance on the steps required to support Anabranch Framework transferability. Adapted from Milat A and Bauman A [50].

Steps	Approach
Step 1	Thoughtful consideration needs to be afforded to the acceptability and relevance of the Anabranch Framework to the context in which transferability is being considered. Scalability of the Anabranch Framework may not be feasible across all contexts and sensitivity will be required to avoid imposing the Anabranch Framework on communities, higher education institutions, service providers and departments with carriage of health professional education in rural contexts.
Step 2	Engage key internal and external stakeholders early in consideration of Framework adoption, adaptation and implementation. Embed collaborative and co-design principles and approaches to ensure the acceptability and relevance of contextualized model development.
Step 3	If a determination is made to advance the potential transferability and implementation of the Anabranch Framework, the development of a strong rationale for this approach will be required, one that takes into consideration the diverse perspectives, strategic objectives, roles and responsibilities and potential impacts for key stakeholders. Share and seek feedback on this rationale as part of the co-design process.
Step 4	Identify the relevant domains, constructs and structural elements of the Anabranch Framework that are of relevance and adaptable to context. Map existing activity against these components and prioritize focus areas for action. Consider an incremental approach to Framework adaptation and model design if required. Estimate the resources required to implement the Framework and model taking into consideration opportunities for resource redirection across key stakeholder agencies and resource scarcity in rural contexts.
Step 5	Through co-design, identify which stakeholders have a role and/or responsibility for key functions for the advancement of Framework transferability and model development, including governance of the initiative and curriculum design. Be prepared for the complexity and time required to work with multiple stakeholders across multiple organizations, and with community representatives to reach a shared consensus.
	Develop a comprehensive implementation strategy, including risk mitigation and considerations for monitoring, evaluating and researching Framework implementation, impact and outcomes. Share the implementation strategy with key stakeholders and potential funding organizations.
Step 6	Consider the strategic, political and structural implications of enacting the Anabranch Framework from multiple perspectives. Identify the levers required to progress, implement, adapt and sustain the investments made and model designed.

## Data Availability

No new data were created or analyzed in this study.

## Supplementary Materials

The following supporting information can be downloaded at https://www.mdpi.com/article/10.3390/healthcare14030406/s1, Table S1: Scholarly contributions to health professional education and practice.

## Abbreviations

The following abbreviations are used in this manuscript:

AF	Anabranch Framework
CIPP	Context, Input, Process, Product
CoP	Community of practice
HPE	Health Professional Education
LIC	Longitudinal integrated clerkship
LIP	Longitudinal integrated placement
RHMT	Rural Health Multidisciplinary Training
TEP	Transformative emancipatory pedagogy
UDRH	University Department of Rural Health
