# Supplementary material for: The Anabranch Framework for the Ruralization of Health Professional Education
Source: Healthcare (Basel). 2026 Feb 5;14(3):406. doi: 10.3390/healthcare14030406 (PMC12897278; doi:10.3390/healthcare14030406)
Supplement: Supplementary file 1 [file healthcare-14-00406-s001.zip › healthcare-4075239-supplementary.pdf]

Table S1. Scholarly contributions to health professional education and practice (Supplementary Table).

Article	Research Objectives	Study Design	Sample	Setting	Intervention-Education Design	Findings	Implications for Practice	Implications for Future Research
Jones, D., Haddadan, G., Dunsmore, M., Williams, A., White, D., Hanniver, J., ... & Randall, S. (2025). Reframing Nurse Education in Rural Australia: Implications for Advancing Longitudinal Integrated Placements	Explore the experiences of final year pre-registration nursing students undertaking a longitudinal integrated placement in rural Australia	Qualitative interviews	Thirteen final year pre-registration nursing students who undertook a longitudinal integrated clinical placement in semester one (n = 7) and two (n = 6) of 2022	Rural primary health care settings in far west NSW and northwest Victoria, Australia	The Extended Nursing Placement Program (ENPP), provides final year pre- registration nursing students with a 20- week LIP. Key rural theories were integrated into the program including health care complexity, community literate health care, community engaged practice, and First Nations health.	Three themes were generated from the data: Experiencing comprehensive care, Making connections and Engaging with complexity. Findings highlight the importance of longitudinal integrated placements for student nurse exposure to comprehensive care, mitigating previous experiences of episodic and fragmented care associated with short duration placements across multiple settings. Placement duration enabled students to establish connections with their peers, health care teams, and the communities in which placements were undertaken. Student exposure to complexity-informed practice extended their capacity to interpret and respond to the broader inequities experienced and complexity as it relates to nursing practice in rural contexts	Complexity theory needs to be embedded in rural curriculum and delivered in a way that is transformative, raising questions that challenge dominant systems of thought. Learning needs to be open, responsive, and relational, to enable students to weave together the complex threads that influence rural health if we are to develop complexity- informed nursing professionals	Not stated
Randall, S., Jones, D. M., Hadaddan, G., White, D., & Einboden, R. (2024). It's all about relationships: Developing nurse-led primary health care in rural communities	Describe the experiences of nursing executives and Registered Nurses (RNs) in designing and transitioning to a nurse-based PHC role	Qualitative case study (interviews and focus groups)	Nursing executives who had oversight of program planning, codesign, and management (n = 3) and PHC RNs involved in service delivery (n = 5)	The PHCRN:SB program was implemented in far west New South Wales (NSW), Australia, in 2017–2018. Collaborating partners engaged in program codesign,	The PHCRN:SB program is a nurse-led PHC model embedded into primary and secondary schools	All participants in this study stated that they were ready for a better work-life balance, professional change and were motivated by the model's upstream health orientations. This change	A slow introduction is necessary for PHC practice, offering the opportunity to appreciate the context holistically, identify individual and population health needs, alternative practices, and	Not stated

Article	Research Objectives	Study Design	Sample	Setting	Intervention-Education Design	Findings	Implications for Practice	Implications for Future Research
				implementation and evaluation included a rural Local Health District Directorate of Nursing and Midwifery (NSW Health), a University Department of Rural Health, and a School Education Network (NSW Department of Education).		happened by cultivating a strategic long-term way of thinking and developing an enhanced skill set aligned to their new PHC roles. The nurses discussed the challenge of moving from an illness to a wellness focus, and how becoming solution focused, rather than problem focused, was a new way of working. Participants commented on needing to slow down in their practice and the time commitments required for them to professionally align themselves to PHC delivery. They needed to put the pieces of the picture together to ensure they were responding in the right way and at the right time, noting that rushing in could be counterproductive	novel approaches, as well as create professional adaptability and flexibility in response to ever-changing and complex circumstances	
Robinson, T., Govan, L., Bradley, C., & Rossiter, R. (2024). Transforming health care delivery: The role of primary health care nurses in rural and remote Australia	Describes the policy context that informs the development of nurse-led models of care, and identifies enablers and barriers that inhibit PHC nurses working to their full scope of practice	Narrative literature review (non-systematic methods)	Not applicable	PHC nurses in rural and remote Australia	The Australian Primary Health Care Nurses Association (APNA) received funding to implement nurse-led clinics as demonstration projects. The clinics enable PHC nurses to work to their full scope of practice, improve continuity of care and increase access to health care in under serviced locations	Governments and the nursing profession have sought to implement new models of nurse-led primary health care and initiatives that enable and expand nurses' scope of practice to include independent practice and prescribing, and transition from specialist to generalist roles	Reforms to nursing employment awards, models of care for complex chronic diseases, and clinical practice guidelines are required to support nurse-led models of care	There is a need to build the evidence base for nurse- led PHC models and the ways in which they can impact on primary health service delivery in rural and remote Australia
Bourke, L., Brand, A., Howard, C., Aitken,	Present a case for the Rural Health	Commentary	Not applicable	Rural Health Multidisciplinary Training	Not applicable	Presents a defence of the impact of RHMT	With equitable access to research funding, RHMT	Not stated

Article	Research Objectives	Study Design	Sample	Setting	Intervention-Education Design	Findings	Implications for Practice	Implications for Future Research
R., Argus, G., Brown, L. J., ... & Versace, V. (2023). Let us not forget what the Romans did do—The impact of RHMT	Multidisciplinary Training (RHMT) program in building an evidence-based for rural health training and development			(RHMT) program funded UDRHs and RCSs across rural and remote Australia		program-funded UDRHs on the basis of:<br>- Relatively young status of UDRHs<br>- Internal workforce challenges<br>- Standards by which rural health research is evaluated<br>- Difficulties in measuring changes in rural workforce recruitment and retention<br>- Broader community and sector benefit of the UDRHs	programs can increase the number and retention of rural researchers, and support collaborations to increase the scope of research activities, and strengthen evidence related to RHMT programs	
Randall, S., White, D., & Dennis, S. (2023). A collaborative primary health care model for children and young people in rural Australia: Explorations of cross-sectoral leader action	Examination of cross-sectoral executive and senior management level system changes made in the design of a collaborative PHC service model for children and young people in rural Australia	Qualitative interviews	One executive and one senior manager were interviewed from each of the three collaborating organisations (n = 6) (Education, Health, and a UDRH)	Seven primary and two high schools within Broken Hill, NSW	The Primary Health Care Registered Nurse: Schools Based (PHCRN:SB) program is a complex, cross-sector, multi-component primary healthcare initiative that supports a wellness model of care for children, adolescents and their families. It seeks to improve health and education outcomes for children and young people. The service is delivered by registered nurses employed by the Far West Local Health District (FWLHD), who are located on nine school campuses	Analysis of the data collected from six participants across three organisations led us to generate three overarching themes: embedded challenges and experimental solutions; building a shared language and understanding; and the role of relationships and trust	Understanding the processes and factors that influence cross-sectoral collaboration is noted as vital to ensure continuing success of projects that run across sectors	Future research can build on studies such as ours by focussing on the successes of systems change to effect improvement in equity for children and young people rather than focussing on outcomes alone
Naden, K., Hampton, D., Walke, E., Pavlovic, S. P., Graham, S., & Jones, D. (2023). Growing Our Own	Describes the activities and learnings of three rural Australian academic health departments in delivering	Non-systematic review of service activities and research outputs	Not applicable	The University of Sydney University Departments of Rural Health located in Broken HIll and Lismore and Rural	The HCAP seeks to provide secondary school students with opportunities to engage with rural, remote and Aboriginal	Identified program enablers include: place- based approaches to health career development; theoretically informed strategies;	The importance of establishing and maintaining trusting and credible relationships with students, schools, health industry	A small body of research has been undertaken on the program; however, challenges exist in accessing

Article	Research Objectives	Study Design	Sample	Setting	Intervention-Education Design	Findings	Implications for Practice	Implications for Future Research
Rural, Remote, and Aboriginal Health Workforce Contributions made, approaches taken, and lessons learnt by three rural Australian academic health departments	the Health Career Academy Program (HCAP)			Clinical School located in Dubbo have invested since 2007, 2017 and 2018, respectively, in the design, adaptation and implementation of the HCAP across far west, western and the north coast regions of New South Wales	health role models to acquire an understanding of the obstacles confronted and the real-world solutions drawn on to address them. The HCAP links students to a number of tertiary pathways, scholarship opportunities, and student support services located within their universities of choice	program leaders who are located within regions with capacity to leverage local relationships and networks across mainstream and Aboriginal health and education sectors; redistribution of department resources to support program delivery; knowledge sharing across departments; built in adaptability to improve program acceptability and efficiency; and program embeddedness within rural academic health departments with carriage of health workforce development and linkages to a range of health services and higher education institutions	and community partners is considered a key feature in enabling program co- design and continuity of engagement. The contextualisation of the program is considered necessary to support place-based responses that are more likely to meet the needs of rural, remote and Aboriginal communities	research funding and the academic staff required to undertake longitudinal evaluations of program impact and workforce outcomes. There is a need to understand which strategies are acceptable and effective if we are to address 'gaps in the Australian rural health research evidence base [which] threaten to leave holes in Australian health policy'
Jones, D., Randall, S., Williams, A., Waters, D., White, D., Haddadan, G., ... & Parr, S. (2022). The Strength of Cross-Sector Collaborations in Co-Designing an Extended Rural and Remote Nursing Placement Innovation: Focusing on student learning in preference to student churning	Describe a cross-sector and multi-university collaboration in co- designing an extended nursing placement innovation in rural and remote Australia	Case report	Not applicable	Far West Extended Nursing Placement Program (ENPP). The program is underpinned by a regional cross- border, cross- sector and multi-university collaboration established in 2019	The program is underpinned by a regional cross- border, cross- sector and multi- university collaboration established in 2019. The ENPP integrates undergraduate nursing and regional health service curriculum, practice exposure and community immersion in a 20- week clinical placement duration for final- year Bachelor of Nursing students	The authors propose that the adoption of collaborative approaches can contribute to reframing student nurse education and the development of a rural- ready nursing workforce. These approaches can provide regions and universities with the opportunity to avoid student churn whilst promoting the attainment of skills required to work, live and thrive in these locations	Extended-duration clinical placements may address the regional- university challenges experienced in student nurse education but may also have the unintended consequence of creating financial burdens for participating students. Program stakeholders are advocating at the state and federal levels for the establishment of funding streams to further support the program and student participation	ENPP student nurse participant critical reflections,<br>regional and academic evaluations and research findings will inform program adaptations to ensure a quality placement experience. A comprehensive evaluation of the pilot phase of the program will also inform program scalability and sustainability within the region
White, D., Jones, D., Harvey, P., Wright, F.,	Describe the establishment of a cross-border	Commentary	Not applicable	The multi-university collaboration brings	Sunraysia Collaboration	Through the establishment of the collaboration	The Sunraysia collaboration demonstrates one	Not stated

Article	Research Objectives	Study Design	Sample	Setting	Intervention-Education Design	Findings	Implications for Practice	Implications for Future Research
Tarrant, L., Hodgetts, L., ... & Livingstone, K. (2022). Competition or Collaboration in Regional Australia: A Cross-Border and Multi-University Approach to Maximising Rural Health Investments, Community Health and Health Workforce Outcomes	and multi-university collaboration in rural Australia - the Sunraysia Collaboration			together the Broken Hill UDRH, The University of Sydney and Monash Rural Nursing and Allied Health (RNAH), Monash University. Broken Hill UDRH is located in the south of Far West of New South Wales and Monash RNAH Mildura is located in North- West, Victoria.		and adoption of co- design approaches, the collaboration has been able to maximise RHMT Programme investments within the region. This has included: the establishment of a well- supported, co-funded and locally embedded academic network; the development of service- learning innovations that enhance the delivery of training to students and access to services of need for communities, including Aboriginal and Torres Strait Islander populations; the provision of structured rural placements that address university curriculum requirements and regional expectations; and student engagement in interprofessional education experiences	approach towards mitigating potential competition between RHMT Programme funded universities within rural and remote Australia. The collaboration is an exemplar of codesign in action providing an alternative approach to address RHMT Programme parameters and regional needs whilst supporting rural- remote health workforce training and education innovations	
Gordon, S., Burns, R., Champion, S., Niutta, L., & Bennett, P. (2022). Compassion and person-centred care Survey development for aged care workers	Assess the reliability of modified questionnaires measuring healthcare worker compassion and patient centred care (PCC)	Survey reliability testing	Aged care workers (n = 25)	Metropolitan Adelaide residential aged care setting	Not applicable	The modified questionnaires were found to have acceptable interreliability and test- retest intra- class correlation for the subscales and overall. However, the investigation also found low Cohen's kappa values between the test and retest responses for the individual items, subscales and overall, and had low inter- class correlation for individual items, indicating poor inter- rater	Not stated	Not stated

Article	Research Objectives	Study Design	Sample	Setting	Intervention-Education Design	Findings	Implications for Practice	Implications for Future Research
						agreement. High inter-item correlation scores also suggest the questions encapsulate overly similar constructs		
Sanford, C., Saurman, E., Dennis, S., & Lyle, D. (2022). What is this about: Let's play this out": the experience of integrating primary health care registered nurses with school learning and support teams	Explore the experiences of Registered Nurses (RNs) and school learning support teams in implementing the School-Based PHC Service (SB-PHCS)	Qualitative focus groups	School learning support team (n = 21) and participating RNs (n = 4)	Six public primary schools, one Catholic primary school and two public high schools. Broken Hill, NSW	The SB-PHCS aims to improve access to health and social services by providing care navigation, kindergarten health screening and health promotion (Table 1). The SB-PHCS employs five Primary Health Care RN full-time equivalents, and was progressively implemented between 2017 and 2019. A key tenet of the program is the co-location of RNs in the schools and their integration with Learning Support Teams (LSTs). LSTs work with students, families, classroom teachers and other professionals to identify and support students with additional learning needs at school	We found that defining the role and working across systems were challenges to program implementation, whereas a collaborative culture, relationship building and flexibility in work processes facilitated the integration of nurses into the school teams	We recommend others embarking on similar initiatives involve key stakeholders early in service development, understand each other's systems and processes, and provide clarity about the new role, but plan to adapt the role to fit the context	Not stated
Walsh, S., Lyle, D. M., Thompson, S. C., Versace, V., Browne, L. J., Knight, S., ... & Jones, M. (2020). The role of national policies to address rural allied health, nursing, and dentistry workforce maldistribution: A scoping review	Introduce the Spinifex Network as a leading national network supporting rural health and medical research and to describe Australian rural health research priority areas	Rapid literature reviews (methods not reported)	Not applicable	Australian rural and remote contexts	Not applicable	Notes several research priorities:<br><br>Food security in rural and remote Australian communities: Prioritising initiatives to improve the healthiness of food environments can be effective; however, the paucity of studies identified in the Australian context	Not stated	The rapid reviews in this supplement sought evidence of the effectiveness of interventions to inform policy and practice and also to identify research gaps in the currently available evidence. The result has been to highlight

Article	Research Objectives	Study Design	Sample	Setting	Intervention-Education Design	Findings	Implications for Practice	Implications for Future Research
						indicates a general under-investment in this area.<br><br>Impact of natural disasters on people living in rural and remote areas: Identified lack of robust evaluation of the various programs and interventions. Examining the short and long term effects of bushfire on children - there is a need for research on effective supportive interventions to improve health and wellbeing outcomes in this population.<br><br>Recruitment and retention of a sustainable rural health workforce: There is a lack of research using well designed longitudinal evaluation designs to evaluate not only the effectiveness of recruitment and retention strategies but also their alignment with policy objectives.<br><br>Global crises affecting people in rural and remote settings: complexity and challenges of tapering opioids in patients with chronic non-cancer pain, identifying several barriers and enablers associated with the current approach		priority areas for targeted research
Jones, D., Lyle, D., McAllister, L.,	Present a conceptual framework that	Conceptual development	Not applicable	Not applicable	Community literacy aims to integrate community	Not applicable	Transitioning community literacy from a concept	Further research is required to develop,

Article	Research Objectives	Study Design	Sample	Setting	Intervention-Education Design	Findings	Implications for Practice	Implications for Future Research
Randall, S., Dyson, R., White, D., ... & Rowe, A. (2020). The Case for Integrated Health and Community Literacy to Achieve Community Responsive Healthcare: An inclusive approach to addressing rural and remote health inequities and community healthcare expectations	describes community literacy, its alignment with health literacy, and its relationship to concepts of community engaged healthcare				knowledge, skills and resources into the design, delivery and adaptation of healthcare policies, and services at regional and local levels, with the provision of primary, secondary, and tertiary healthcare that aligns to individual community contexts. A set of principles is proposed to support the development of community literacy. Three levels of community literacy education for health personnel have been described that align with those applied to health literacy for consumers. It is proposed that community literacy education can facilitate transformational community engagement. Skills acquired by health personnel from senior executives to frontline clinical staff, can also lead to enhanced opportunities to promote health literacy for individuals		into practice requires further discussion within the healthcare sector, the development of educational resources, and the inclusion of community voices to determine its acceptability and relevance within rural and remote Australian contexts	validate, and evaluate the three levels of community literacy education and alignment to health policy, prior to promoting its uptake more widely
Aggar, C., Mozolic-Staunton, B., Scorey, M., Kemp, M., Lovi, R., Lewis, S., ... & Thomas, T. (2020). Interprofessional primary healthcare student placements: Qualitative findings	Investigate the attitudes, knowledge, skills, and behaviour of students towards interprofessional collaboration and teamwork following participation in an Inter-Professional Education (IPE) program	Qualitative focus groups	62 students participated in this phase of the research. Focus group participants included undergraduate nursing (n = 32) and allied health (n = 30) students. Allied health students included speech pathology,	The School of Health and Human Sciences at Southern Cross University developed an IPE placement in primary healthcare settings (rural/remote and international) for undergraduate students in the disciplines of nursing,	At a regional Australian university, an interprofessional experiential clinical placement experience for undergraduate healthcare students in primary healthcare settings was developed, implemented and evaluated. The IPE program	Students regarded the placement as a rich learning environment and reported positive experiences in interprofessional collaborative competencies. Major themes included: overall perceptions and attitudes, scope of practice, improved	According to best practice methods, students should engage in IPE learning prior to their placement experience. Effective placement preparation enables students to learn together in multi-professional groups and delivers structured academic	Despite students' assertions that their newly acquired IPE competencies would transfer to their future practice as health professionals, it is difficult to determine whether this is the case. Future research is required

Article	Research Objectives	Study Design	Sample	Setting	Intervention-Education Design	Findings	Implications for Practice	Implications for Future Research
from a mixed-method evaluation			occupational therapy and podiatry. Seperate focus groups were conducted for students in Cambodia, Vietnam, and rural Australia<br><br>The mean age of the students was 30 years (SD 8.1, range 19–46), and the majority were female (79%), with no previous IPE experience (89%).	speech pathology, occupational therapy and podiatry. All students were enrolled in a unit of study underpinned by Primary Healthcare and Health Promotion. The teaching and learning undertaken in this unit provided the academic context for the IPE placement	was implemented in 2017 in primary healthcare settings in rural and remote Australian communities and internationally (Cambodia and Vietnam)	teamwork skills, future practice, and placement preparation	content centred on teamwork and collaboration	that focuses on a longer follow-up period to explore whether collaborative skills are sustained into future practice. Research in the future could also benefit from conducting pre-placement focus groups to compare the level of change in collaborative competencies with post-placement data
Aggar, C., Mozolic-Staunton, B., Lovi, R. J., Scorey, M., Kemp, M., Walker, T., & Lewis, S. (2020). An interprofessional clinical placement in a primary healthcare setting: a pilot study	Evaluate the impact of a interprofessional education (IPE) clinical placement in a PHC setting on students' perceptions of interprofessional education	Single-arm (pre-post test) intervention study	19 students who completed both a pre, and post-placement questionnaire, taken from 63 students invited to participate	The School of Health and Human Sciences at Southern Cross University provided an IPE clinical placement opportunity in primary health settings (rural/remote and international) for undergraduate students (speech pathology, occupational therapy, podiatry and nursing) enrolled in a unit of study underpinned by Primary Health Care and Health Promotion	Placements spanned three to six weeks, and included three to twenty students, from at least three different disciplines at each site. Students participated in three pre-placement workshops, aimed to increase the students' understanding of placement expectations, student and facilitator roles and responsibilities, as well as orientation to the placement sites. The structure of the IPE placement was underpinned by the principles of primary health care, including community participation in defining and implementing health needs and health promotion activities.6 Daily meetings and debriefs to discuss challenges, opportunities, and	There was a significant increase in competency and autonomy and understanding of roles over time. There was no significant increase in the reported perception of need for interprofessional cooperation, however there was a significant increase in actual interprofessional cooperation<br><br>This study has demonstrated that an IPE clinical placement in a primary healthcare setting is a valuable medium for delivering motivating interprofessional learning experiences. It can provide opportunity to practice teamwork skills and develop a deeper understanding of the roles, responsibilities and perspectives of other disciplines	Most of the pre-placement academic content that students were exposed to focused on health promotion and primary healthcare. Exposing students to comprehensive IPE theory, a comprehensive introduction to the core competencies of interprofessional teamwork, and workshop activities that focus particularly on interprofessional collaboration may strengthen students' preparation for their IPE placement experience and their perception of the need for interprofessional cooperation	This was a successful pilot program that warrants further development and research to include longer term follow up of students' perceptions towards IPE and analyse whether collaboration and teamwork skills obtained during the IPE primary healthcare experience are transferrable to future professional practice

Article	Research Objectives	Study Design	Sample	Setting	Intervention-Education Design	Findings	Implications for Practice	Implications for Future Research
					community health needs were conducted with experienced university clinical facilitators. Consequently, a wide variety of health education was delivered, including benefits of exercise, menstruation hygiene, hand hygiene, nutrition, sun safety, oral hygiene, eye care, communication milestones education, social skills development, swallowing and mealtime management. Students were also required to develop a health promotion initiative that involved an interprofessional team working on intersectoral collaboration			
Sanford, C., Saurman, E., Dennis, S., & Lyle, D. (2020). "We're definitely that link": the role of school-based primary health care registered nurses in a rural community	Explore the case support role of Registered Nurses (RNs) working in a school-based PHC service (SB-PHCS) during the first 18 months of program implementation, from the perspective of teachers working in learning support teams and the RN workers	Qualitative focus groups and interviews	School learning support team (n = 21) and participating RNs (n = 4)	Seven primary and two high schools within Broken Hill, NSW	The SB-PHCS places Local Health District-employed RNs in schools to provide support for students with chronic and complex issues, health screening for kindergarten students and health promotion services. The Service is underpinned by principles of community engagement, family-centred care, trauma-informed care, integrated care and a life phases approach to student health, and integrates RNs with existing	Two main categories described the RN role: the 'care navigator' category is specific to the support provided for individual students, whereas 'the link' is a conceptual theme encompassing the broader role of the RN as an agent within the health and education systems.	This study suggests that embedding RNs within learning support teams, rather than providing services to families independent of school-based supports, enhances family engagement with both health and education and improves the flow of information between systems.	Future studies should document this evolution and capture families' and other care providers' experiences of the service. Although the present study provides examples of successful navigation for individual students, comprehensive evaluation of the SB-PHCS is required to assess model effectiveness, including cost effectiveness, in improving health and education outcomes

Article	Research Objectives	Study Design	Sample	Setting	Intervention-Education Design	Findings	Implications for Practice	Implications for Future Research
					school learning support teams			
Jones, D., Ballard, J., Dyson, R., Macbeth, P., Lyle, D., Sunny, P., ... & Sharma, I. (2019). A Community Engaged Primary Healthcare Strategy to Address Rural School Student Inequities: A Descriptive Paper	Describe the design and implementation of the School-Based PHC Service (SB-PHCS)	Case report	Not applicable	Cross-sector collaboration between school education, a local health district, and a university department of rural health	The PHCRN:SB strategy was intentionally designed to respond to the unique rural community context, was informed by community experiences and expectations of healthcare for children, adolescents and their families, and seeks to align nursing practice and service provision to the principles of primary healthcare. Five new full-time primary healthcare registered nursing positions have been established to deliver health promotion programs, enhance healthcare access through the earlier identification of needs and service activation, and contribute to service coordination and integration for children and adolescents already experiencing complex/chronic conditions. These positions are co-located on primary school campuses and transition into secondary school settings with the intent to establish and maintain healthcare and health professional relationship consistency and continuity	Challenges experienced in attracting suitably qualified registered nurses to the positions. Additional investments have been required to support the appointed PHCRNs in transitioning from hospital-centric and acute care practice to schools-based and primary healthcare practice. Substantial resource, time and education investments have been directed towards nurse preparation for primary healthcare practice, including the enrolment of all nurses in post-graduate primary healthcare coursework	Not stated	Three key research streams associated with the strategy have been identified for exploration: (1) service recipient impacts and outcomes; (2) cross-sector systems impacts and; (3) implications for the establishment and maintenance of a rural Australian primary healthcare nursing workforce

Article	Research Objectives	Study Design	Sample	Setting	Intervention-Education Design	Findings	Implications for Practice	Implications for Future Research
Edwards, J., Hooper, D., Rothwell, G., Kneen, K., Watson, J., & Saurman, E. (2019). A Nurse Practitioner-led Community Palliative Care Service in Australia – a narrative report	Describe the background, design, and function of the San Community Palliative Care Service (SanCPCS) and identify essential elements of the service model	Qualitative case study	'Key informants' - type and nature not specified	The San, is the largest acute-care private and not-for-profit hospital providing acute surgical, medical, and obstetric care in NSW	The San Community Palliative Care Service (SanCPCS) operates in four stages: (1) Triage, (2) Weekly multi-disciplinary meetings and regular home visits, (3) Identification of deteriorating and terminal phases, and (4) Death	The SanCPCS supports both the patient and their family, allowing patients to die in the place of their choosing. The service also provides freedom of choice and supporting personal control of a life-limiting illness has the dual advantage of improving general health and personal wellbeing, as well as alleviating pressure on the hospital and healthcare systems	Possible limitations with a nurse practitioner-led service include consideration for succession planning and leave cover. Other issues include access to other providers and systems that also affect care and financial stability for service delivery in a community setting are challenges that are still to be resolved	Not stated
Held, F. P., Roberts, C., Daly, M., & Brunero, C. (2019). Learning relationships in community-based service-learning: a social network analysis	Describe the network of learning relations that allied health students recalled at the end of a six-week community-based service learning program	Social network analysis using exponential random graph models (ERGMs)	OT and SP students participating in a school-based service learning program (n = 10)	The Allied Health in Outback Schools Program (AHOBSP), developed through a fifteen-year collaboration between the community of Broken Hill, the Broken Hill University Department of Rural Health (BHUDRH), several universities, and other key stakeholders including government	The context for this research study is the "Allied Health in Outback Schools Program" (AHOBSP). For the cohort of AHP students in our study (occupational therapy (OT) and Speech therapy) the program included regular classes and workshops at BHUDRH, and the students' main role was to conduct lessons in local primary schools and kindergartens to further the children's lingual and motor development. In teams of two or three students had to plan, prepare, conduct and evaluate classes. Team membership was fixed for each school for the duration of the placement, but different combinations of students teamed up in	Data was available from a complete cohort of 10 students on placement in a network of 69 members, providing information on 680 potential learning relations. Students engaged in similar ways in the domains of clinical knowledge, procedural skills, and professional development. Learning relations with academic supervisors were significantly more likely. Students reported reciprocal learning relations with peers. This effect was absent in learning networks about complex determinants of health (including socio-economic and cultural factors). Instead, local administrative staff were significantly more often the source of	Understanding the structure of student learning relationships through a social network analysis helps identify potential points of leverage to create and improve more targeted education and training strategies for allied health students to optimise the use of community-based service-learning programs. Clinical educators could promote social learning by preparing students to more readily engage with each other and with members of the community	Future research could adapt the methodology of our social learning network analysis to local contexts. This may provide quantitative measures of the multiple factors underpinning learning in clinical placements and potentially relate them to measures of student learning outcomes

Article	Research Objectives	Study Design	Sample	Setting	Intervention-Education Design	Findings	Implications for Practice	Implications for Future Research
					different schools. School teachers were present during each of their classes. Students lived in shared housing facilities with students of other disciplines during their placement	learning about the local contextual factors		
Jones, D. M., McAllister, L., & Lyle, D. M. (2018). Rural and Remote Australian Speech-Language Service Inequities: An Australian Human Rights Dilemma	Call to action for how health and education sectors can work together to ensure children have accesss to essential speech pathology services	Non-systematic review of service activities and research outputs	Not applicable	Speech and Language services in rural and remote Australia	Within rural and remote Australian contexts, university departments of rural health, key stakeholders in rural and remote health service design and workforce development, are investing in the formation of civically engaged community–campus partnerships and SLP service-learning programs as an alternative approach to addressing service inequities, educational reform and workforce shortages. Since the inception of these Australian innovations in 2009, over 1000 rural and remote children have received services from over 200 SLP students. These SLP students have been exposed to alternative, civically engaged and schools-based models of healthcare delivery	Not applicable	Not stated	Not stated
Jones, D., McAllister, L., Dyson, R., & Lyle, D. (2018). Service-Learning Partnerships: Features that	Describe features that promote transformational and sustainable community engaged health partnerships	Qualitative focus groups and interviews	Participants were those involved in the school-based service learning clinic, including school principals	Local partnership between school education, health sectors and the BHUDRH to explore potential service	Broken Hill primary school principals approached the Broken Hill University Department of Rural Health	Features that support university-health-school partnerships include:<br>- responding to community need	Findings from this study support the need to re-orientate health sectors from 'delivering services that meet their needs' to	Health partnerships are concerned with service outcomes, however, an exploration of service impacts

Article	Research Objectives	Study Design	Sample	Setting	Intervention-Education Design	Findings	Implications for Practice	Implications for Future Research
promote transformational engagement and sustainable rural and remote health partnerships and services	and services in rural and remote Australian locations		(n = 7) and senior managers (n = 2) from local facilitating agencies. Campus participants included allied health students (n = 10) and academics (n = 2), one rurally located with student supervision responsibility and one metropolitan located with a strategic partnership role	solutions to allied health service inequities experienced by children in Broken Hill, NSW	(BHUDRH) to voice their concerns about the lack of paediatric allied health services and the impact of this on children's development. In response, the BHUDRH facilitated a local partnership between school education, health sectors and the BHUDRH to explore potential service solutions	- offering services of value<br>- drawing from community leadership and innovation<br>- working within trusting relationships<br>- seeking consistency in service provision<br>- sharing knowledge and using this to adapt joint programs	service delivery that responds to what people expect of health care for themselves, their families, and communities. If we can achieve this re-orientation then we have greater capacity to establish community engaged health partnerships and services that are reciprocal and mutually beneficial.	on school children was beyond the scope of this study. This study explored the experiences of participants engaged in one rural and remote Australian health partnership and service-learning program limiting generalisability of findings. However, the service-learning program has been adapted and implemented in other rural and remote locations and research is currently being undertaken to explore impacts of participation
Kirby, S., Held, F. P., Jones, D., & Lyle, D. (2018). Growing health partnerships in rural and remote communities: What drives the joint efforts of primary schools and universities in maintaining service learning partnerships?	Explore factors which enabled and sustained an intersectoral learning program between universities and schools, focusing on organisational partnerships	Mixed methods - qualitative interviews and focus groups, and social network analysis	Focus group/interview (n = 49) and survey respondents (n = 39), including placement hosts, university hosts, and university sources from across Broken Hill, Geraldton, and Katherine	School-based paediatric speech pathology (SP) service learning programme in Broken Hill (NSW)—with comparison to additional rural/remote sites which adopted the model – Geraldton (Western Australia) and Katherine (Northern Territory)	A cohort of SP students worked in schools each term to provide a rolling programme, tracking pupil progress for a year and handing over to the next cohort. The service learning programme spans three sectors: primary school education, tertiary undergraduate education and health	Factors supporting partnerships were long-term, work and social relationships, commitment to community, trust and an appetite for risk-taking. This study found that strong long-term partnerships had developed communities to support cross sectoral programmes. Partnerships are more likely to prosper in rural and remote communities where personal and social interactions overlap, because individuals in the partnership are more likely to trust their fellow workers in the partnership and more likely to take risks to maintain the	An important 'take-home message' for academic institutions wanting to adopt service learning is the value of local community relationships forged over many years, strengthened by work and social interactions between individuals with a desire to improve community outcomes	Similar studies with other programmes at a range of other locations would help to clarify our findings. Other limitations are the lack of rigorous evidence on the impact of the service learning programme on school pupil communication outcomes and on undergraduate student learning. Both are areas for future research

Article	Research Objectives	Study Design	Sample	Setting	Intervention-Education Design	Findings	Implications for Practice	Implications for Future Research
						partnership for the good of their community		
Kirby, S., Lyle, D., Jones, D., Brunero, C., Purcell, A., & Dettwiller, P. (2018). Design and delivery of an innovative speech pathology service-learning program for primary school children in Far West NSW, Australia	Describe the design and implementation of speech pathology student-led clinics (service learning) and report service impact of the program	Evaluation of service activity and outcomes data (using student case notes and Wilkins Screen of Articulation and the Screen of Communication Scores	Primary school children in Broken Hill NSW, who were identified as requiring Speech Pathology assessment (n = 101) following health screening	Primary schools in Broken Hill, NSW	Each school term, a new group of six speech pathology students provided services for 5–7 weeks. For each child, they provided screening and assessments, and made plans for treatment if indicated. Assessment after screening confirmed the screening findings and indicated the appropriate therapy.<br><br>Each new cohort of speech pathology students was informed by handover notes from the previous student group, and continued to screen, assess and treat children during the program cycle. Student activities were supervised by the BHUDRH speech pathology academic in close liaison with teachers, student learning and support officers, and school principals to ensure the program fitted with the smooth running of the school curriculum. Student supervision included individual and group face-to-face sessions with the academic supervisor at the BHUDRH and teleconferencing with the academic	Analysis of the outcome data demonstrated improvements in communication impairments for approximately one-quarter of the children<br><br>At the end of the program cycle of one year's therapy, 24 children (24%) were discharged having reached age-appropriate skills, 56 (55%) were given communication goals to address residual problems and 12 (12%) were referred for ongoing care from community-based or hospital speech pathologists. There were no statistically significant differences in treatment outcomes for the service learning program between children with mild and moderate delays/disorders. Only one of the 12 children with a severe delay/disorder was discharged having reached an age-appropriate level; the rest required ongoing access to speech pathology interventions	These findings show that clinically based allied health service-learning programs are feasible and can be sustainable in the Australian setting. The success of these programs is also linked to the community–academic partnerships that underpin the student-led clinic model in schools. Clinical-based service-learning programs can address gaps in service availability under specific circumstances and deliver acceptable and accessible care while working effectively in collaboration with established services	For the Broken Hill service model, additional data are needed about the intensity and duration of treatment by all modalities, the goals of therapy and goal attainment to determine whether the program has achieved its full potential for measurable clinical improvement. Further research should also examine the impact of the program on children's classroom engagement and participation, and learning, as well as on educational outcomes such as basic numeracy and literacy. The low sensitivity of the screening tools may have limited the extent to which false positives were identified. This is a possible limitation. Another limitation is that other confounding factors that may have accounted for improvements in children's communication were not identified

Article	Research Objectives	Study Design	Sample	Setting	Intervention-Education Design	Findings	Implications for Practice	Implications for Future Research
					at the student's university when necessary. Students were usually in the final year of their course			
Kirby, S., Lyle, D., Jones, D., Brunero, C., Purcell, A., & Dettwiller, P. (2018). Embedding Public Health Advocacy into the Role of School-Based Nurses: Addressing the Health Inequities Confronted by Vulnerable Australian Children and Adolescent Populations	Describe the advocacy roles of school-based nurses and the barriers and enablers affecting their advocacy work	Commentary / narrative review (non-systematic methods)	Not applicable	School-based PHC nursing	Not applicable	Not applicable	Appropriate additional qualification would expand the potential of SBNs to process and use population-level data, such as school screening data, in ways that would make inequities more difficult to ignore, refocus care to include population-level practice and integrate local knowledge of inequities into efforts to promote service and policy action	Not stated
Lyle, D., & Greenhill, J. (2018). Two Decades of Building Capacity in Rural Health Education, Training and Research in Australia: University Departments of Rural Health and Rural Clinical Schools	Describes the contribution of University Departments of Rural Health (UDRHs) and Rural Clinical Schools (RCSs) to the development of rural health and the rural health workforce	Non-systematic review of service activities and research outputs	Program data and research outputs from Australian UDRHs and RCSs as of 2018	Australian UDRHs and RCSs	The UDRHs and RCSs now provide academic and organisational support for both undergraduate and graduate-entry students in the health disciplines. The academic centres deliver cross-cultural, inter-professional and simulation training for students and resident health practitioners	As a result, university Departments of Rural Health and Rural Clinical Schools have established a substantial geographical footprint covering most of the rural and remote populations and regions across Australia. They have a large distributed rural clinical academic workforce that exceeds 1300. Medical student numbers on long-term placements have increased threefold from inception to 1200 annually. Allied health and nursing numbers doubled over 10 years to 4000 in 2013 and are projected to double again by 2018. In 2013, they published 363 peer-reviewed papers –	To maintain and expand this workforce, universities and other employing agencies will need to adapt how they manage the employment of rural academics to take account of those factors that influence the recruitment and retention of rural health practitioners. This will also require ongoing investment in developing the next generation of rural clinical academics and succession planning systems	Not stated

Article	Research Objectives	Study Design	Sample	Setting	Intervention-Education Design	Findings	Implications for Practice	Implications for Future Research
						half of which specifically addressed rural and/or remote health issues. High levels of intention to practise rurally and uptake of rural and remote practice following exposure to rural training have been reported, especially for medicine		
Humphreys, J., Lyle, D., & Barlow, V. (2018). University Departments of Rural Health: Is a national network of multidisciplinary academic departments in Australia making a difference?	Evaluate the role and contribution of University Departments of Rural Health (UDRHs) to teaching, research and health service performance in rural and remote Australia, prior to expansion funding announced in 2015	Mixed-methods - descriptive analysis of program data and qualtiative interviews with UDRH staff	Data were obtained from annual UDRH key performance indicator (KPI) reports to the Australian Government Department of Health detailing undergraduate- and graduate-entry domestic student clinical placement activity (of duration ≥2 weeks) between 2009 and 2013, and research publications for 2013	Australian University Departments of Rural Health	See article for overview of teaching and research activities across Australian UDRHs	Common features across all UDRH student programs included availability of cross-cultural, interprofessional and simulation training, orientation of students to placements, and UDRH-managed accommodation. Other features varied by context across the network. The UDRHs generated 220 peer-reviewed papers in 2013 of which 86% were applied research and 40% addressed some aspect of rural and/or remote health. UDRHs also contributed academic input to many significant regional projects that aim to develop new models of care, improve service access, support better-trained health professionals, or build capacity in organisations and communities	The data presented in the article could assist in benchmarking performance for the new Rural Health Multidisciplinary Training Program	Information is lacking on the extent to which this investment is promoting the uptake of rural or remote practice after graduation or improving health outcomes
Roberts, C., Daly, M., Held, F., & Lyle, D. (2017). Social learning in a longitudinal	Explored the relationship between student learning, student perceptions of preparedness for practice, and	Qualitative interviews	Eighteen (n =18) medical students from three consecutive cohorts who undertook their longitudinal clinical	Broken Hill University Department of Rural Health (BHUDRH)	The Broken Hill Extended Clinical Placement Program (BHECPP), where clinical learning occurs in	Through the theoretical lens of social learning systems, two major themes were identified; connectivity and preparedness for	Supporting students to understand the significance of connectivity constitutes a challenge for learning and teaching,	Future research could undertake further analysis of the social interactions influencing student learning

Article	Research Objectives	Study Design	Sample	Setting	Intervention-Education Design	Findings	Implications for Practice	Implications for Future Research
integrated clinical placement	student engagement, in the context of a rural longitudinal integrated clinical placement (LIC)		placements in Broken Hill during 2010 (n = 6), 2011 (n = 5), and 2012 (n = 7)		community and hospital settings. All students are hosted by a general practitioner (GP) supervisor for the duration of their stay, and work for an average of four sessions each week in the practice, some of which employ a parallel consulting setting. Students live in accommodation hubs with other student health professionals, also undertaking clinical placements, and are encouraged to engage in broader community activities (sport, music, community service, volunteering etc.)	practice. Connectivity described engagement and relationship building by students, across formal and informal learning experiences, interprofessional interactions, social interactions with colleagues, interaction with patients outside of the clinical setting, and the extent of integration in the wider community. Preparedness for practice, reflected students' perceptions of having sufficient depth in clinical skills, personal and professional development, cultural awareness and understanding of the health system, to work in that system	for academics and clinical teachers as well as those with responsibility for development of the placement at the community level. Additional support may be required for the minority of students who demonstrate little agency	by developing the methodology of finding meaningful quantitative measures of learning developed from qualitative data using social network analysis
Lyle, D., Saurman, E., Kirby, S., Jones, D., Humphreys, J., & Wakerman, J. (2017). What do evaluations tell us about implementing new models in rural and remote primary health care: Findings from a narrative analysis of seven service evaluations conducted by an Australian Centre of Research Excellence	Describes key findings from seven Centre of Research Excellence (CRE) in Rural and Remote PHC service evaluations to better understand what made these primary health care (PHC) models work where they worked, and why	Narrative literature review	Fiveteen (n = 15) articles reporting on seven CRE service evaluations of different PHC models, published between 2012 and 2015	PHC service reforms across rural and remote settings in Western Australia, Victoria, Northern Territory, and NSW	Four of the seven evaluations reported on community-based services: two innovative comprehensive PHC services—one in a remote Western Australian Indigenous community and the other in a small rural community in Victoria, and one aged care service and one social and emotional wellbeing service—in remote Indigenous communities from the Northern Territory and Kimberley region of Western Australia.	Three different contexts for PHC reform were evaluated: community, regional and clinic based. Three themes were identified from the evaluation reports: enabling changes to PHC delivery that resulted in more appropriate PHC services, processes that support services to improve access to PHC, and requirements for service adaptation to promote sustainability in changing internal and external environments	The review highlighted that shared decision-making, negotiation and consultation with communities, tailored to context, is important and should be used to promote feasible strategies that improve access to PHC services	There is a growing need for service evaluations to include reporting on the feasibility, acceptability and fit of successful PHC service models within context to provide evidence for local dissemination, adaption and implementation strategies

Article	Research Objectives	Study Design	Sample	Setting	Intervention-Education Design	Findings	Implications for Practice	Implications for Future Research
					Regional service reform: Two regional outreach service models were evaluated: provision of diabetes cycle of care to patients through the Royal Flying Doctor Service (South Eastern Section) network of fly-in fly-out clinics in far west New South Wales (NSW), and a virtual outreach, telehealth service established by a local health district (LHD) to improve access to emergency mental healthcare across western NSW.<br><br>Clinic-based reform: The final service evaluation reported on modifications to a clinic process that introduced patient-led scheduling of appointments for routine mental health practice in a remote township of the Northern Territory to enhance the efficient and effective use of limited healthcare resources			
Jones, D., McAllister, L., & Lyle, D. (2016). Community-Based Service-Learning: A Rural Australian Perspective on Student and Academic Outcomes of Participation	Understand the impacts and outcomes of participation in a community-campus partnership and service-learning program, from the perspective of participating allied health students and academics	Qualitative focus groups and interviews	Occupational therapy (n = 4) and speech pathology (n = 6) students, who represented one student cohort undertaking its placement in one school term in 2014<br><br>Allied health	Allied Health in Outback Schools program, initiated in 2009 in response to concerns raised by community leaders, namely primary school principals, about the detrimental educational, health, and social outcomes for	A cross-sector partnership was established between local health and school education sectors, and the Broken Hill University Department of Rural Heal, a rural department of the University of Sydney. Representatives from the	Broad codes were developed and then collapsed into two themes: catalysts for program participation and civic impacts of participation	Health students, academics, and professionals need to access rural community knowledge to enhance<br>their capacity to effectively engage with communities. The development of community-literate health professionals	A lack of evidence of the impact of participation in community-based service-learning for faculties represents an identified gap in the literature

Article	Research Objectives	Study Design	Sample	Setting	Intervention-Education Design	Findings	Implications for Practice	Implications for Future Research
			academics—one in a rural setting who had over three years of responsibility for the direct education and supervision of students engaged in the service-learning program, and one in metropolitan setting who had contributed to the development of the program and had a continuing strategic partnership and programmatic role	children experiencing developmental delays who were unable to access allied health services.	university's Faculty of Health Sciences, with responsibility for allied health education, contributed to the development of a community-based and interprofessional service-learning program<br><br>Cohorts of final-year occupational therapy and speech pathology students from four Australian universities participate in the program across the four school terms. Students provide screening, assessment, therapy, and referral services, drawing on the work of previous student cohorts to inform service delivery<br><br>Supervision approaches include discipline and interprofessional supervision, student peer supervision, and school teacher supervision		is just as important as the development of health-literate consumers, and reciprocal investment at the policy, funding, education, and practice levels is required to achieve community-literate health systems and professional outcomes	
Jones, D., McAllister, L., & Lyle, D. (2016). Challenging remote community deficit perspectives: an Australian insight into the role of these communities in the design of their health services and development of their health workforce	Describe the formation of a community-campus health partnership and the development of a service learning program, and to develop a greater understanding of the impacts of participation on community and campus participants	Qualitative focus groups and interviews	School principals (n = 12), senior managers from health and university agencies (n = 2), allied health students (n = 10), and allied health academics (n = 2)	Partnership governed by health—a local health district of the NSW Ministry of Health, far west NSW school education—NSW Department of Education, a University Department of Rural Health (the Broken Hill UDRH), and an external university faculty, the	Partners collaboratively developed an allied health service-learning program. The program aligned senior OT and SP students' learning to the provision of allied health services to school-aged children. Services are now provided across three regional communities and twelve school	The role of community partners in initiating the partnership was described and conditions associated with remote contexts and health sector failures were identified catalysts. Service and learning adaptation, partnership commitment and service consistency, service acceptability and accessibility,	Health and higher education sectors need to have a greater level of responsiveness to locally developed and led health innovations, and student education that may challenge traditional supervision and training models	Not stated

Article	Research Objectives	Study Design	Sample	Setting	Intervention-Education Design	Findings	Implications for Practice	Implications for Future Research
				University of Sydney Faculty of Health Sciences.	campuses. Six SP and four OT students undertake an inter-professional service-learning placement for periods of six to eight weeks across the four school terms	and community investment in remote health workforce development were identified impacts		
Wakerman, J., Humphreys, J., Bourke, L., Dunbar, T., Jones, M., Carey, T. A., ... & Murakami-Gold, L. (2016). Assessing the Impact and Cost of Short-Term Health Workforce in Remote Indigenous Communities in Australia: A Mixed Methods Study Protocol	To identify the impact of short-term health staff on: the workload, professional satisfaction, and retention of resident health teams in remote areas, (ii) the quality, safety, and continuity of patient care, and (iii) service cost and effectiveness	Study protocol	Not applicable—study protocol	The study sites include remote clinics managed by the Northern Territory (NT) Department of Health, and remote Aboriginal community-controlled health services	Not applicable	Study protocol only	Not stated	Not stated
Jones, D., McAllister, L., & Lyle, D. (2015). Interprofessional Academic Service-Learning in Rural Australia: Exploring the impact on allied health student knowledge, skills, and practice. A qualitative study	Explore the perspectives of stakeholders (school principles, senior healthcare managers, allied health students and academics) regarding the impact of a school-based service learning model	Qualitative interviews and focus groups	Occupational therapy (n = 4) and speech pathology (n = 6) students, who represented one student cohort undertaking a school-based placement in one school term in 2014<br><br>Sample also included academic staff (n = 2), including one rural academic with direct supervision of students and one metropolitan academic with a strategic role in the program	The Allied Health in Outback Schools Program (AHOBSP) commenced in 2009, responding to concerns raised by primary school principals on the detrimental impacts for children who were unable to access allied health services	Serial cohorts of OT and SP students from four universities, now undertake placements across four school terms. Student to supervisor ratios are 4:1 for OT, and 6:1 for SP. Students, as interprofessional teams, under the supervision of discipline and interprofessional qualified clinicians, provide screening, assessment and therapy services for school children with mild to moderate needs across twelve school sites and three regional communities. Children with more complex needs are referred to hospital clinicians.	Broad codes were developed and collapsed into three key themes: previous interprofessional practice exposure, program supervision model, and interprofessional practice impacts	Not stated	Although this study describes valuable IPP student learning outcomes service-learning is equally concerned with outcomes experienced by service recipients. Additional research is required in this area if we are to meet the intent of service-learning, that of reciprocal benefit and value of service and learning activity

Article	Research Objectives	Study Design	Sample	Setting	Intervention-Education Design	Findings	Implications for Practice	Implications for Future Research
					Approximately 150 children access these services annually. Additional interprofessional program elements include a five day intensive induction in Broken Hill, weekly clinical and professional reflection sessions and mid- and end of placement focus group evaluations			
Jones, D., McAllister, L., & Lyle, D. (2015). Stepping out of the shadows: Allied health student and academic perceptions of the impact of a service-learning experience on student's work-readiness and employability	Describes the impact of participation in a rural Australian service-learning program on student and academic perceptions of work-readiness and future employability	Qualitative interviews and focus groups	Occupational therapy (n = 4) and speech pathology (n = 6) students, who represented one student cohort undertaking a school-based placement in one school term in 2014<br><br>Sample also included academic staff (n = 2), including one rural academic with direct supervision of students and one metropolitan academic with a strategic role in the program	Primary and secondary schools in Broken Hill, NSW	The Allied Health in Outback Schools Program (AHOBSP) was informed by the principles of service-learning and experiential learning. Serial inter-professional, OT and SP, student cohorts undertake placements across the four school terms. Students participate in an intensive five day orientation program and weekly professional reflection sessions facilitated by the BHUDRH with services being delivered in pre-schools and primary school settings. Students, with same-discipline, as well as inter-professional supervision, from qualified OTs and SPs, provide screening, assessment and therapy as inter-professional groups for children with mild to moderate developmental delays. Children identified with	Study participants identified a number of beneficial learning outcomes acquired through their participation in the program including insight into rural community contexts, complex and interconnected relationships, planning and organisation skills and enhanced self-confidence	Service learning may provide students, universities and the health industry with an additional approach that can contribute to the development of generic work-readiness attributes	Additional research is required to explore program impact on greater numbers of participating students and academics. Comparative studies are also required that explore learning outcomes between program participants and non-participating peers

Article	Research Objectives	Study Design	Sample	Setting	Intervention-Education Design	Findings	Implications for Practice	Implications for Future Research
					complex delays are referred to hospital clinicians for further assessment. Supervision is provided through scheduled face-to-face contact throughout the students' 'working week' and via email, SMS and telephone			
Jones, D., Lyle, D., Brunero, C., McAllister, L., Webb, T., & Riley, S. (2015). Improving Health and Education Outcomes for Children in Remote Communities	Explores the challenges and implications of a novel cross-sectoral approach to health services delivery and health workforce development	Case report	Not applicable	The Allied Health in Outback Schools Program (AHOBSP), delivered within far west NSW	The adopted approach saw cohorts of final-year speech pathology and occupational therapy students from The University of Sydney undertaking their clinical placement experiences in primary school settings in far west NSW across three school terms. Prior to their placement, participating students took part in a discipline-specific, five-day comprehensive preparation for practice program on site in Broken Hill. The students, under the supervision of qualified discipline-specific clinicians, provided screening, assessment and therapy for children identified with mild to moderate needs. Children identified with complex developmental delays and emotional and social needs were referred to hospital clinicians for more intensive	Notes key challenges to developing a health workforce to improve child health outcomes:<br>- policy and funding environment<br>- parental involvement in services<br>- acceptance of service-learning as a legitimate pedagogical approach<br>- health workforce development and higher education tailored to the needs of rural healthcare practice<br>- development of cross-sector collaborations	There is a clear message in the US that the university sector has a social responsibility mandate. How or if this is interpreted and translated into practice within the Australian context in the current policy and funding environment will impact on the relevance of higher education institutions across the broader Australian population and remote subpopulations	If Australia is to adopt service-learning as a meaningful approach to pre-registration education for future health professionals, then theory development and practice implementation that account for Australia's unique geography and vast population spread, as well as our health and education systems, needs to be at the core of this movement. Robust research that explores the impact of service-learning for service recipients, communities, participating students and higher education institutions is urgently required to identify the efficacy of Australian responsive models

Article	Research Objectives	Study Design	Sample	Setting	Intervention-Education Design	Findings	Implications for Practice	Implications for Future Research
					assessment. Supervision in the initial stages of model development was supported by academics and clinicians employed through The University of Sydney and the FWLHD			
Kirby, S., Moore, M., McCarron, T., Perkins, D. & Lyle, D. (2015). Nurse led diabetes in remote communities	Test the feasibility of providing a nurse-led annual cycle of diabetes care in a remote location and explore factors that patients indicated were important in diabetes self-management	Mixed methods - patient clinical outcomes (bivariate correlations) and qualitative patient interviews	A total of 21 patients took part in the pilot study. Three patients were from a remote township with outreach services and no resident nursing or medical services, 8 were from a remote township with resident nursing services and outreach services, and 10 were from a town with resident nursing and medical services	The study sites, which were all in New South Wales, were a remote township with outreach services and no resident nursing or medical services, a remote township with resident nursing services and outreach services, and a town with resident nursing and medical services. Both remote townships were provided with visiting outreach nursing and medical services	The nurse-led cycle of diabetes care was developed from evidence-based protocols implemented by the chronic disease nurse (CDN) under the medical supervision of GPs in a shared care model. The same nurse visited each patient over the course of a year, from February 2013 to February 2014. Patients' weight and girth were measured at the beginning and at the end of the pilot, and advice was given about medications, diet, weight loss and exercise. In addition, lifestyle changes were reviewed at quarterly intervals by the CDN under GP supervision, thereby conforming with the diabetes management guidelines of the Royal Australian College of General Practitioners	The program resulted in a modest but significant mean reduction in HbA1C levels of 0.7% but no significant differences in weight or glomerular filtration rate. Medication regimen, living alone, age, sex and patient location had no significant influence on patient outcomes<br><br>The estimated average total care per patient, including travel time, was 10.9 hours in the annual cycle of care for an average drop in HbA1C 15.57 hours for a drop in HbA1C cost in nursing hours per 1% drop in HbA1C therefore A$242.45<br>Factors important in motivating patients toward self-management were trust in the CDN, CDN support, personal resources and making lifestyle management a priority	Special training on the social, psychological, emotional and motivational aspects of diabetes is needed to improve the uptake of self-management	Service models research has focused on rural and regional settings rather than on remote settings. More research is needed to test models of care that produce improved outcomes; this will secure the evidence base for interventions that are effective in remote communities
Thomas, S. L., Wakerman, J., & Humphreys, J. S. (2015).	Determine consensus among rural and remote health experts	Delphi consensus-building methodology	Delphi group (n = 28), taken from a subset of a larger Delphi group	For the purpose of this study, remote communities were described as	Not applicable	The population thresholds for core PHC services provided by a resident	These findings ascertaining the population thresholds at which core PHC	Not stated

Article	Research Objectives	Study Design	Sample	Setting	Intervention-Education Design	Findings	Implications for Practice	Implications for Future Research
Ensuring equity of access to primary health care in rural and remote Australia - what core services should be locally available?	regarding the population thresholds at which core PHC services should be provided by resident (local) health workers		of 39 experts that had been engaged in previous research on the core PHC services<br><br>Selection of individuals was based on their knowledge, experience and length of time working in the field of rural or remote health. Attention was paid to ensuring wide representation from areas of policy, the academic community, clinical practice and consumer representation. All states and territories were represented and members of key rural and remote health organisations were included	communities with small populations, located at a considerable distance from larger centres, usually in sparsely populated regions. These communities often have a high proportion of resident Indigenous Australians and a high degree of isolation (ASGC categories 4 and 5). Rural communities, on the other hand, referred to those relatively larger and/or less isolated communities located in more densely populated regions, which tend to be closer to larger centres where more comprehensive services may be available, such as hospitals and visiting or resident specialists (these are non-metropolitan rural communities not in ASGC 4 or 5)		worker were less in remote communities compared with rural communities. The population threshold for 'care of the sick and injured,' was ≤100 for remote compared with 101–500 for rural communities. For 'mental health', 'maternal/ child health', 'sexual health' and 'public health' services in remote communities the population threshold was 101–500, compared to 501–1000 for rural communities. Principles underpinning implementation included the fundamental importance of equity; consideration of social determinants of health; flexibility, effective expenditure of resources, tailoring services to ensure consumer acceptability, prioritising services according to need, and providing services as close to home as possible	services would be best provided by resident health workers provide some guidance to policy makers and service planners tasked with the allocation of scarce resources for the provision of PHC services	
Kumar, K., Jones, D., Naden, K., & Roberts, C. (2015). Rural and remote young people's health career decision making within a health workforce development program: a qualitative exploration	Explore the factors shaping rural and remote youths' career choices following participation in a health workforce development program	Qualitative interviews and focus groups	Thirty-three participants (n = 33) in the BHRHCAP, including current and past secondary students, school principles, teachers, career advisors, parents, university students, and clinicians from local health services	Broken Hill is a mining city in the far west of outback New South Wales with a population of approximately 20 000 people. Its health workforce is made up of a large fly-in, fly-out workforce, and higher and vocational education and training opportunities available for	The Broken Hill Regional Health Career Academy Program (BHRHCAP) engages secondary school students in years 7–12 in various health career activities, with the healthcare system and professionals within it. Each 1-day academy involves experiential small group activities related to	Findings show that personal, contextual and experiential factors impact on rural and remote young people's health career decision-making. Personal factors encompassed young people's personal goals, aspirations and confidence to engage in career decision-making; contextual factors related to the	Increasing the breadth of exposure to a range of careers allied to health (such as healthcare assistants, healthcare administrators) would serve to engage the subset of rural and remote youth who may not be vocationally oriented and further widen participation	Not stated

Article	Research Objectives	Study Design	Sample	Setting	Intervention-Education Design	Findings	Implications for Practice	Implications for Future Research
				young people in the region are limited	health promotion and health literacy, information sharing about health careers, education pathways and options, and visits to various hospital and community-based healthcare sites including Aboriginal health services, the Royal Flying Doctor Service, private practice and aged care	BHRHCAP program structure as well as the geographical, social, cultural and economic landscape in which young people are located and make their career decisions; and experiential factors were related to having access to role models or influential others		
Daly, M., Roberts, C., Kumar, K., & Perkins, D. (2013). Longitudinal integrated rural placements and learning: a social learning systems perspective	Explore the pedagogical and socio-cultural underpinnings of students learning within a longitudinal, integrated, community-based rural placement	Qualitative interviews	Medical students (n = 16), General Practitioners (n = 8), and other clinicians (n = 10) participating in the Broken Hill Extended Clinical Placement Programme (BHECPP)	The specific longitudinal placement studied was the Broken Hill Extended Clinical Placement Programme (BHECPP). It is hosted by a university Department of Rural Health (UDRH)	Longitudinal Placement of 6–12 months duration. The programme involves a collaboration between institutions of higher education and their larger communities (local, regional, state, national, global) for the mutually beneficial exchange of knowledge and resources in a context of partnership and reciprocity. Learning occurs concurrently and is continuous and immersive. For example, three 8-week blocks in surgery, medicine and community would be run as one long 24-week integrated block	The process of connectivity helps explain how students access and cross the boundaries between learning spaces and develop a more complex sense of professional identity	Students should be taught how to manage the tensions associated with traversing the boundaries of multiple learning spaces, which may be better managed by improving student and staff orientation to the underpinning philosophy and activities of the placement programme and providing ongoing professional development for supervisory staff	This paper did not examine the potential experiences of student peers in other clinical settings, but a comparative study looking at experiences in metropolitan and regional settings is planned to further explore this aspect
Gladman, J., & Perkins, D. (2013). Training Australian General Practitioners in Rural Public Health: Impact, desirability and adaptability of	Explored the impact, desirability and suitability of a hybrid problem-based learning (PBL) model for public health training in rural General Practitioner (GP) training	Mixed method - pre-training cross-sectional survey and post-training qualitative interviews	General Practice (GP) trainees (n = 17) and tutors (n = 4)	Rural General Practitioner (GP) training	The aim was to build knowledge in clinical and holistic care, cross-cultural awareness, rural public health, and to develop critical thinking, communication and teamwork. The broad	Six main themes emerged: experience; PBL impact; learning modalities; educational needs; educational expectations; and educational planning.	Opportunities for improving the delivery of PBL sessions are described, including providing more complex and relevant cases, and condensing training/education content	A modified hybrid model of PBL, based on the approach used in the study, warrants further examination

Article	Research Objectives	Study Design	Sample	Setting	Intervention-Education Design	Findings	Implications for Practice	Implications for Future Research
hybrid Problem Based Learning					title 'outback itches' was chosen to direct thinking from clinical practice to population health. The scenario involved a 7-year-old Aboriginal boy who had red, itchy hands, wrists and feet for several days. He lived with his extended family and was brought to the GP by his Grandmother			
Perkins, D., & Daly, M. (2013). What is the evidence for clinical placements in under-served areas?	Explorations of strengths and weaknesses of community placements in under-served rural areas, from the perspective of medical students and supervisors	Narrative literature review (non-systematic methods)	Literature review - no record of eligible/reviewed articles	Clinical placements in Australian rural settings	Not applicable	Supervisors are drawn from general medical practitioners in under-served areas who report personal and professional rewards from participation and hope that their participation will add to the resident workforce and relieve pressure of work in the future. They report some anxiety about how their teaching relates to the medical curriculum and this may reflect medical student concerns about upcoming examinations. Key issues such as the translation of intention to practice rurally into rural career choices may be influenced by early rural experience and rural origin, but may also be affected by factors such as the availability of internship positions and broader opportunities for career progression in disadvantaged locations	Training, development and support of supervisors is needed, as well as motivated students	More research is needed using ethnographic and case study methods to understand how, why and when these models work and how they can be better designed to benefit disadvantaged communities

Article	Research Objectives	Study Design	Sample	Setting	Intervention-Education Design	Findings	Implications for Practice	Implications for Future Research
Bennett, P., Jones, D., Brown, J., & Barlow, V. (2013). Supporting rural & remote primary health care placement experiences increases undergraduate nurse confidence	Describe the impact of a structured and comprehensive educational clinical placement experience on undergraduate nurse's levels of confidence in the areas of primary health care and culturally knowledgeable practice	Mixed methods - single arm (pre-post test) intervention study (self-rated confidence) and qualitative focus groups and interviews	Undergraduate nurses participating in the Primary Health Care Intensive Programme (PHCIP) in far west New South Wales between 2006 and 2008 (n=31)<br><br>The study cohort consisted primarily of female students (84%), with the average age of participants being 30 years. All undergraduate nurses lived on the mid-north to north coast of NSW, Australia. One participant was lost to medium term (3 month post-placement) follow-up.	The study occurred between 2006 and 2008 in far west New South Wales (NSW)	The Primary Health Care Intensive Programme (PHCIP) provides undergraduate nurses with the opportunity of a 4–8 week fieldwork experience in rural and remote PHC sites across the far west NSW. Students participate in a comprehensive 5-day orientation programme in Broken Hill prior to commencing their onsite placement within broken Hill or partner communities in the region. Undergraduate nurses then travel to host sites for clinical placement. BHUDRH nurse academics and support staff make weekly contact to students and host sites. This allowed early identification of potential issues and to provide support, problem solving, advocacy and clinical support to students and host site staff as needed. After placement, undergraduate nurses return to BHUDRH for a one-day de-brief/clinical supervision session and completion of the post-placement evaluation tool.	The findings of this study identified factors such as a supported/structured orientation and an extended immersion placement experience can influence an undergraduate nurse's confidence in delivering culturally competent, and person-centred care in a rural/remote PHC context in a positive way. Findings suggest that the support of 'host' site staff in welcoming undergraduate nurses to the community and to the work environment is invaluable in ensuring a supported learning experience	Given that rural/remote clinical experience and Indigenous health clinical experience are not a required component of Bachelor of Nursing (BN) curricula in NSW (Nurses and Midwives Board of New South Wales 2003) it is unlikely that students have had any exposure to these opportunities as an undergraduate. Any opportunity that arises to provide urban undergraduate nursing students with a rural/ remote clinical experience should be actively pursued	The long-term follow-up of participants of the PHCIP will provide researchers with a greater understanding of the long-term impact of this experience on participant career choices
Bennett, P., Barlow, V., Brown, J., & Jones, D. (2012). What do New Graduate	Identify the factors in an integrated, community-engaged rural placement that may	Qualitative interviews	Student participants were 24 medical students, in their final 1–2 years of medical	Medical placements at the Broken Hill UDRH	The Broken Hill Extended Clinical Placement Program (BHECPP) uses an integrated	Students and clinicians identified three key factors contributing to P4P: enhanced opportunity for	It is important to select students for the program who are more proactive, flexible and seek out	Further exploration of the longitudinal experiences of the rural and remote clinical

Article	Research Objectives	Study Design	Sample	Setting	Intervention-Education Design	Findings	Implications for Practice	Implications for Future Research
Registered Nurses want from jobs in Rural & Remote Australian Communities	contribute to preparedness for practice (P4P) from the perspective of students and clinicians		school, on rural placement at Broken Hill UDRH between 2010 and 2012. Participants also included eight general practitioners, 10 hospital clinicians and three community health nurses.		curriculum, with concurrent learning blocks over a 6–12 month placement period. Within the program, there are three distinct clinical practice communities: general practice; hospital and remote community care, each characterised by different learning opportunities and experiences such as parallel consulting in general practice and team collaboration in the hospital. The students are hosted by a general practice and spend two to four half-day sessions there each week, with the remaining time spent in the base hospital, as well as four weeks on remote placement	clinical learning; personal and professional development and developing cultural skills.	experiences. Some students prefer a more structured learning environment. An increased openness to learning opportunities might be achieved through better orientation, highlighting initiative and a broad range of strategies to recognise and maximise learning opportunities.<br><br>There is also a need to better support rural clinical supervisors provide a high quality learning environment	supervisor exploring continuity and quality of remote supervision would be useful
Daly, M., Perkins, D., Kumar, K., Roberts, C., & Moore, M. (2013). What factors in rural and remote extended clinical placements may contribute to preparedness for practice from the perspective of students and clinicians?	Explore the experiences of PHC nurses in rural and remote NSW who are required to provide palliative care as part of their generalist role	Mixed methods - cross sectional survey and qualitative interview (sequential design)	Registered Nurses (n = 30) and Enrolled Nurses (n = 4) participated in the survey research, of which 10 were purposefully sampled for qualitative interviews	14 rural and remote sites in this study were part of the former Far West Area Health Service (NSW).<br><br>The PHC nurses were based in a variety of facilities. Some were small Multi Purpose Services (MPS) with a nursing clinic, no in-patient beds and weekly Royal Flying Doctor Service clinics. Others were larger MPS with some in-patient beds	Not applicable	Rural and remote community PHC nurses working in Far West NSW are experienced and juggle multiple roles as part of their generalist work. They regard palliative care as important, although infrequent cases affect feelings of clinical competence. They report a variety of attitudes and preferences to palliative care.<br><br>Some have a strong preference for palliative care nursing, while others only	A high level of commitment to patients and their communities was a strong motivator for these nurses who provided palliative care under difficult conditions. How local health services equip and support them is critical if palliative care services are to be sustained in rural and remote communities	A question for further research is how preferences for palliative care relate to competency and how these preferences might influence patient care. Other questions include how to support nurses who have a particularly bad experience in providing palliative care; how to help them deal with issues of grief and loss recognising that some may be providing care

Article	Research Objectives	Study Design	Sample	Setting	Intervention-Education Design	Findings	Implications for Practice	Implications for Future Research
				and a resident GP. There was one larger regional hospital with resident GP services and a general physician		look after palliative care patients because it is a job requirement. Most nurses agreed that the PHC role description in the Role Delineation Framework reasonably reflects their work, yet their feelings of being equipped to provide palliative care is demonstrated in different levels of competence and confidence for particular elements of care. They face challenges associated with geographical and professional isolation, lack of resources and barriers to education and support, while the emotional nature of working with people from small communities who are dying can be very difficult		for family members or close friends; and how to provide appropriate relief to prevent burnout whilst simultaneously working within flexible models of care that facilitate short-term after hours support for dying patients
Bolte, K., Bennett, P., & Moore, M. (2012). ENRICHing the rural clinical experience for under graduate health science students: A short report on interprofessional education in Broken Hill.	Describes the development, implementation, and value of the Enhanced Rural Inter-Professional Cultural Health (ENRICH) Program	Qualitative focus group	Not applicable	The Broken Hill University Department of Rural Health (BHUDRH)	ENRICH offers IPE that enhances and integrates the clinical skills of health science students. It is designed to 'value-add' to the students' experience through sessions that blend clinical and non-clinical experiences across a range of disciplines. The sessions are held weekly, mostly occupying half a day. ENRICH employs simulation and other group learning activities that are facilitated by local	A focus group with an independent facilitator and student satisfaction has been evaluated at the completion of each session. Feedback reported as almost uniformly positive	Not stated	Not stated

Article	Research Objectives	Study Design	Sample	Setting	Intervention-Education Design	Findings	Implications for Practice	Implications for Future Research
					and external experts. Didactic techniques have been kept to a minimum in response to student evaluation			
Moore, M., Bolte, K., & Bennett, P. (2012). Innovative training for rural medical students	Describe the Enhanced Rural Inter-Professional Cultural Health (ENRICH) program	Qualitative program evaluation	Not applicable	The Broken Hill University Department of Rural Health (BHUDRH)	The Enhanced Rural Inter-Professional Cultural Health (ENRICH) programme. The programme was conceived as a 'value-adding' component that complements the curricula of the three home universities of the medical students. It has evolved to include students from other health sciences in discussing a broad range of rural and cultural health issues. The content of ENRICH sessions is placed in a rural context wherever possible. This is achieved by inviting people from the local community to contribute, and by framing clinical problems in a specific local context. The interprofessional aspect of the sessions is highlighted. The core attendance at sessions is by medical students, but students from other disciplines attend according to the topic	Results of evaluations for semester 1 in 2011 showed that for all sessions 90 per cent or more of students felt that their learning objectives were met, the workshop was appropriate and enjoyable, and that they would recommend the workshop to others. A qualitative evaluation of the programme was performed at the end of semester 1. The students felt that ENRICH was a positive and distinctive experience, and the interprofessional contact was valued. Some students wanted more hands-on sessions, some want more clinical sessions, and some want more art. There was general consensus that 'overall it balances out'	The ENRICH programme puts issues of life and practice in a rural community firmly on the agenda of BHUDRH students. It gives them an experience of the depth of a rural community that they might otherwise not be aware of and promotes teamwork through interprofessional education. It is another strategy for encouraging students to return to rural work	Not stated
Bennett, P., Barlow, V., Brown, J., & Jones, D. (2012). What do	Explore and describe the needs of new graduate registered nurses	Narrative literature review (systematic methods)	Literature review - no record of eligible/reviewed articles	Literature examining the needs of nurses working in Australian	Not applicable	Three main themes emerged: expectations, support and workloads.	A structured programme for new staff that reduces losses and improves	Unexplored areas of research for new graduate registered nurse

Article	Research Objectives	Study Design	Sample	Setting	Intervention-Education Design	Findings	Implications for Practice	Implications for Future Research
New Graduate Registered Nurses want from jobs in Rural & Remote Australian Communities	in a rural and remote (R&R) setting within Australia			rural and remote contexts		Themes reflected enablers and disablers of retention of nurses as they transition from student role to new graduate practice. New graduates are aware of their limitations as new practitioners. There is an expectation that their employers will provide a supportive learning environment for them to gain the skills necessary to become proficient and valuable employees	retention rates should be considered for trial within Australian and in particular in the rural and remote context	experiences include issues of social inclusion, particularly within the community setting, and the relocation for employment of new graduate registered nurses to towns/communities away from existing family and friends. There is a need for further research on this topic that is inclusive of the expectations of new graduate registered nurses, nurse unit managers, nurse educators and employers, and is conducted using a multisite longitudinal design
Roberts, C., Daly, M., Kumar, K., Perkins, D., Richards, D., & Garne, D. (2012). A longitudinal integrated placement and medical students' intentions to practice rurally.	Exploration of the impact of an integrated placement on medical students' attitudes towards rural practice. Specifically, the study explored how longitudinal integrated rural placements impacted on the personal, contextual, and experiential factors that shape students' interest, choice, motivations, and intentions to return to rural practice after graduation	Qualitative interviews and focus groups	n = 28<br><br>Included medical students, general practitioners, and community health staff	Broken Hill, NSW.	The Broken Hill Extended Clinical Placement Programme (BHECPP) is a integrated multi-university programme in rural and remote medicine aimed at senior medical students in the last 2 years of medical school. Clinical learning occurs in community and hospital settings	The study provides insights into the way in which students' intentions to practise rurally are influenced by the informal curriculum within a longitudinal integrated rural placement. Such placements serve to shape evolving understandings of rural practice, including notions of professionalism and professional identity	Students' sense of themselves as active and valued partners in service delivery and patient care could be further enhanced by developing formal curricula that include educational opportunities such as service-led and interprofessional learning activities	The development of social constructivist theory that provides a framework for understanding more about how the rural learning environment can best be constructed to promote the achievement of not only education outcomes, but also of outcomes related to service delivery and rural workforce provision
Jones, D., Grant-Thomson, D., Bourne, E., Clark, P., Beck, H.,	Description of a university-school service learning program	Case report	Three groups of final year SP students (n = 17) who completed	The Broken Hill University Department of Rural Health (BH UDRH)	Student-run clinics in primary schools. The development relied on non-	A total of 231 primary school aged children, including 167 from	Not stated	Not stated

Article	Research Objectives	Study Design	Sample	Setting	Intervention-Education Design	Findings	Implications for Practice	Implications for Future Research
& Lyle, D. (2011). Model for Rural and Remote Speech Pathology Student Placements: Using Non-Traditional Sites and Partnerships	delivering school-based speech pathology services		their fieldwork placement during 2010	operates a successful multidisciplinary rural clinical placement program in far western New South Wales. In Broken Hill, local primary school teachers and parents had raised concerns about the lack of paediatric speech pathology services and the impact this was having on educational attainment	traditional partnerships with school education, a commitment by speech pathologists from the Area Health Service to allocate time for clinical supervision and work by BH UDRH staff to engage academic partners from a feeder university, recruit students and manage the placements.<br><br>Students worked in pairs running clinics at local primary schools supervised by local speech pathologists. Clinical activity varied with each placement. This first group of the year focused on screening kindergarten children while subsequent rotations screened other children referred by parents or teachers. The students delivered speech pathology interventions for children with straightforward problems, assisted speech pathologists in complex cases and referred to associated services if required. Individual student needs were closely monitored and tailored levels of clinical and non-clinical supervision/ support developed to enhance participant experiences. Students	kindergarten (93% of enrolments) were assessed in 2010. Fifty-eight per cent of kindergarten children had a speech pathology intervention. Furthermore, the number of new referrals on the speech pathology service waiting list has decreased from 250 clients in September 2009 to eight in September 2010. Both formal and informal feedback from speech pathology students, teachers, parents and health staff about the program has been positive and three students have already returned for an 'adult' placement in Broken Hill		

Article	Research Objectives	Study Design	Sample	Setting	Intervention-Education Design	Findings	Implications for Practice	Implications for Future Research
					also participated in the local inter-professional learning program. The curriculum requirements for the placement were determined and monitored by academic staff from the Faculty of Health Sciences, University of Sydney and delivered collaboratively on-site			
Bennett, P., Brown, J., Barlow, V., & Jones, D. (2010). What do new graduate registered nurses say they want from jobs in remote communities?	Gain insight into the long-term needs of new graduate registered nurses in rural and remote contexts	Qualitative focus groups	Eighteen (n = 18) new graduate registered nurses who commenced a 12-month new graduate program under a university (BHUDRH) and health service partnership from 2008-2009	Graduate nursing program delivered by the Broken Hill UDRH	12-month nursing graduate program	New graduate registered nurses have realistic expectations about their limitations as new practitioners and expect their employers to provide a supportive learning environment for them to learn the skills (clinical and nonclinical) necessary for them to become proficient in their profession	Not stated	Researchers acknowledge the results represent a small sample size and propose to move to a formal stage, with the larger study being inclusive of new graduates, nurse unit managers and nurse educators
Perkins, D., Hamilton, M., Saurman, E., Luland, T., Alpren, C., & Lyle, D. (2010). The GP Clinic - Promoting access to primary health care for mental health service clients	Evaluate an innovative rural service offering comprehensive primary health care for mental health service clients	Mixed methods - service data and qualitative interviews	Mental health care providers (n = 15) and adult mental health clients (n = 120) operating wtihin a mental health service within a rural General Practice (GP) clinic	A rural NSW community	A monthly joint Community Mental Health Team (CMHT)—General Practitioner clinic at a local general practice. The CMHT was responsible for organising client appointments and assisting clients to attend (including providing transport if needed). They also accompanied clients during their consultation. No co-payments were charged and details of the consultation were recorded in the general practice and CMHT client records	Between 38 and 54 individuals (19–27% of all CMHT clients) accessed the GP Clinic each 6 months. Continuing use of the GP Clinic for primary health care services was recorded for 40% of clients. Proportionately more clients with psychotic disorders relied on the GP Clinic for continuing services compared with clients with mood disorders and other diagnoses. The providers said that the GP Clinic was not a	There might be opportunities to improve the collaborative model through greater involvement of the psychiatrists in the collaboration and the development of formal models of shared care to further improve physical and mental health care, and through better support for the GP in developing a special interest in mental health care	Questions remain as to whether this model will benefit patients in the longer term by improving health outcomes and changing their use of other health services. A further study is planned to address these questions and to further examine the systemic components of collaboration

Article	Research Objectives	Study Design	Sample	Setting	Intervention-Education Design	Findings	Implications for Practice	Implications for Future Research
						complex service to develop and that it was straightforward to run. They indicated that it was successfully integrated into the normal operations of the general practice and financially viable, making use of existing funding mechanisms only.<br><br>The GP Clinic was credited with improving access for mental health clients to general practice services. Its main purpose was to support those clients who did not have a GP and those unlikely to seek out primary health care services themselves. The CMHT indicated that the GP Clinic was acceptable to their clients and that no one referred had refused to use the service		
Webster, S., Lopez, V., Allnut, J., Clague, L., Jones, D., & Bennett, P. (2010). Undergraduate nursing students' experiences in a rural clinical placement	Explores aspects of rural placements that are effective in engaging students in the learning process	Single arm (pre-post test) intervention study	Eight (n = 8) second-year nursing students from the Australian Catholic University, North Sydney, in partnership with the BHUDRH, who participated in a 4 week rural placement in far western New South Wales.<br><br>There were five female and three male nursing students who participated in this	A primary health care intensive elective facilitated by Broken Hill University Department of Rural Health (BHUDRH)	4 weeks' rural placement in far western New South Wales. As students live in rural communities for the placement, they were able to experience rural life and professional practice first-hand which helps to contextualise their clinical experience	Students reported an increased level of confidence following their clinical placement. The major themes that emerged from the questionnaires relating to the students perceived important issues were support for learning, feeling part of the clinical team, feeling valued for their contribution to patient care, and obtaining diversity of clinical experience. These findings indicated that positive	Students will likely benefit from improvements to pre-placement preparation and placement support, opportunities to learn about culturally appropriate healthcare, and improving supervisor staffing and access to technology	Not stated

Article	Research Objectives	Study Design	Sample	Setting	Intervention-Education Design	Findings	Implications for Practice	Implications for Future Research
			placement. The ages ranged between 19 and 53 years with a mean age of 34 years. Half of the group had never experienced rural life before.			experiences for students are more likely to be related to their actual clinical experience and how valued and supported the students felt rather than the physical aspects of a placement		
Davies, G. P., Perkins, D., McDonald, J., & Williams, A. (2009). Integrated primary health care in Australia	Describes how Australian PHC is currently integrated and assesses the likely impact of current and proposed sector reforms	Policy review (non-systematic)	Not applicable	Primary health care in Australia	Primary health care links communities to first contact heath care, facilitates access to other health and related services and coordinates care for those with complex and chronic care needs. To perform these tasks well, primary health care itself needs to be well integrated, internally (e.g., between general practice and other primary health care services) and externally (e.g., with hospitals or community care)	Reforms being considered by the federal government include bringing primary health care under one level of government with a national primary health care policy, establishing regional organisations to coordinate health planning, trialling voluntary registration of patients with general practices and reforming funding systems. If adopted, these could greatly improve integration within primary health care	Not stated	Not stated
Powell Davies, G., Williams, A. M., Larsen, K., Perkins, D., Roland, M., & Harris, M. F. (2008). Coordinating primary health care: an analysis of the outcomes of a systematic review	To identify strategies used for coordinating care within PHC or between PHC and other services, and to describe their effectiveness in relation to health, patient satisfaction, and economic outcomes	Narrative literature review (systematic methods)	Literature relating to PHC coordination, published between January 1995 and March 2006, and based in countries of Australia, the US, the UK, NZ, Canada, and the Netherlands (n = 80)	Studies were included if they were from Australia, Canada, New Zealand, the United Kingdom, the United States or The Netherlands; were experimental studies (randomised controlled trials and quasi-experimental studies) or evaluation studies (trials, pilots, intervention studies, controlled before and after, comparative studies); and focused on	Arrangements to improve communication between service providers, including case conferencing (56 studies)<br><br>Using systems to support care coordination, including care plans, shared decision support, patient-held or shared records, shared information or communication systems, and a register of patients (47 studies)	Six types of strategy were identified at patient/ provider level, falling into two groups: (i) communication and support for providers and patients, and (ii) structural arrangements to support coordination. These were broadly consistent with existing typologies. All were associated with improved health and/or patient satisfaction outcomes in more than 50% of studies, and interventions using multiple strategies	With the increasing burden of complex and chronic care, PHC needs a strong foundation for care coordination. In Australia, it seems unlikely that continued incremental change will create the relationships between service providers, between providers and patients, and across the systems that are required to support effective coordination of care. New gains will require more fundamental reforms of the	Not stated

Article	Research Objectives	Study Design	Sample	Setting	Intervention-Education Design	Findings	Implications for Practice	Implications for Future Research
				coordination of care involving PHC	Structured arrangements for coordinating service provision between providers, including coordinated or joint consultations, shared assessments, and arrangements for priority access to another service (37 studies)<br><br>Providing support for service providers, including support/ supervision for clinicians, training (joint or relating to collaboration), reminders, and arrangements for facilitating communication (33 studies)<br><br>Structuring the relationships between service providers and with patients, including co-location, case management, multidisciplinary teams or assigning patients to a particular primary health care (PHC) provider (33 studies)<br><br>Providing support for patients, including education (joint or relating to sharing care), reminders, and assistance in accessing PHC (19 studies)<br><br>Organisational level Joint planning, funding and/or management of a program or service (7	were more successful than those using single strategies	overall governance of the PHC sector, the arrangements for funding care and its coordination, and the relationship between citizens and their PHC services	

Article	Research Objectives	Study Design	Sample	Setting	Intervention-Education Design	Findings	Implications for Practice	Implications for Future Research
					studies)<br><br>Formal agreements between organisations (3 studies)<br><br>System level Changes to funding arrangements (1 study)			
Martin, E., & O'Reilly, S. (2007). Consumer collaboration in health education	Describe the development of an education workshop delivered to health professionals interacting with persons experiencing mental illness	Qualitative case study	Not applicable	Consumer support group and network within remote NSW (Broken Hill)	The workshop is a continuing collaborative project bringing together different perspectives with a common goal. The perspectives of the traditional mental health worker and the emerging professional role of consumer advocate are woven together to teach workers about the impact of health care practice on the individual experiencing mental illness in order to improve care. The crucial element of the workshop is the personally delivered story of the consumer and linking the 'lived experience' to theoretical practice models	To test the validity of our style of presentation to health staff, we used a short, generic evaluation form. The results were positive. All participants found the topic interesting or very interesting. The area of most interest was unanimously the personal stories and their integration with the topics. There was no area of least interest. Suggestions for improvement concerned the length of the session and increasing the interactive activities. Other comments included thinking that other hospital staff would benefit and that a better introductory session would help. All participants commented on the usefulness of the workshop and the benefit they had received from attending	Not stated	Although it is possible to describe progress of the workshop in regard to stated objectives, it is not possible to measure the achievement of program goals until further workshops are held with more disciplines involved. The effects on the consumer of recounting the 'lived experience' publicly would benefit from further research
Perkins, D., Larsen, K., Lyle, D., & Burns, P. (2007). Securing and retaining a mental health workforce in	Identify strategies that local managers can use to optimise recruitment and retention of	Mixed methods: qualitative interviews and cross-sectional survey (using	Forty-one (n = 41) staff members were interviewed from the four community mental health teams including	The former Far West Health Area of New South Wales	Not applicable	Overall job satisfaction was high (68%). Key attractors were rural lifestyle and environment. Family reasons, the field	Strategies to recruit and retain staff must take account of personal needs and aspirations. While there is room for state	One or more cohort studies, perhaps in association with University Departments of Rural Health, might

Article	Research Objectives	Study Design	Sample	Setting	Intervention-Education Design	Findings	Implications for Practice	Implications for Future Research
Far Western New South Wales	mental health staff in rural locations	a job satisfaction scale)	managers, team leaders, team members and interns, which represented 85% of the workforce. The majority (28) were experienced workers with five or more years experience. However, 11 respondents were interns (n = 6) or workers with less than five years experience (n = 5) and two were agency staff			of work and the rural lifestyle were factors that keep staff in their positions. Some mentioned the desire to achieve professional goals and see projects completed. Reasons for extended intention to stay were: greater career opportunities; a desire to complete professional goals; extension of positions; and personal factors. The most common reason for leaving was better career opportunities. Other reasons included: changes to personal commitments; heavy workloads or burnout; service management; and workplace politics	strategies to improve employment incentives, there is also considerable scope for local managers to improve the design and attractiveness of jobs	enable more generalisable learning about securing and retaining a mental health allied workforce
Lyle, D., Klineberg, I., Taylor, S., Jolly, N., Fuller, J., & Canalese, J. (2007). Harnessing a University to address rural health workforce shortages in Australia	Determine whether an institution-led (university) response add value to existing faculty and program-based efforts to improve rural health education	Cross-sectional survey	Staff responsible for course coordination within the faculties of Dentistry, Medicine, Nursing and Midwifery, and Pharmacy (n = 4); and eight disciplines of the Faculty of Health Sciences (n = 8) within the University of Sydney	Faculties of Dentistry, Medicine, Nursing and Midwifery, and Pharmacy; and eight disciplines of the Faculty of Health Sciences within The University of Sydney	Not applicable	Of the two educational strategies associated with future rural employment, more progress had been made with rural placements, which was offered by all but one of the health courses. Common to most placements was some form of preplacement orientation, support during the placement and the opportunity for student feedback and evaluation. In view of the emerging evidence that the quality of the rural placement is an important determinant of future rural employment, it seems reasonable to	Incorporating rural workforce outcomes as a shared strategic objective across the health faculties has been an important step in obtaining institutional level support for a coordinated approach at the University of Sydney	Not stated

Article	Research Objectives	Study Design	Sample	Setting	Intervention-Education Design	Findings	Implications for Practice	Implications for Future Research
						assume that enhancing the academic and general support provided would improve the student experience		
Lyle, D., Morris, J., Garne, D., Jones, D., Pitt, M., Walker, T., & Weston, R. (2006). Value adding through regional coordination of rural placement for all health disciplines: the Broken Hill experience	Describes nine-year experience of the Broken Hill University Department of Rural Health (BHUDRH), delivering a regional program in far western New South Wales	Qualitative case study	Case study of the University of Sydney's Broken Hill University Department of Rural Health (BHUDRH)	The Australian Government's University Departments of Rural Health Program, established in 1997, has been responsible for setting up regionally coordinated rural placement programs for students from all health disciplines in each state and territory. The BHUDRH is located in far western NSW and operates as a multidisciplinary academic unit of the University of Sydney	Broken Hill has had a long tradition of accepting medical, nursing and allied health students from across Australia. When the BHUDRH was established in 1997, it took responsibility for organising student placements within the region and building on existing arrangements by establishing a single coordination point for the whole region and all the health organisations—a one-stop shop for student placements	The long lead times in building an academic team and the complexity of organising placements for students from different disciplines and multiple universities restricted early efforts to broaden the educational contribution to the student program. However, in 2002 the BHUDRH was approached to establish a local campus of the University of Sydney's School of Rural Health. Local educators were recruited to deliver approximately six hours of structured teaching each week to stage 3 medical students from the School of Rural Health in addition to ward-based teaching and lectures accessed by videoconference. This involvement has subsequently expanded in 2005 to include students for the stage 4 community rotation, with local academics modifying elements of the rotation to focus on remote health practice and primary health care and by placing students with the Royal Flying Doctor Service.	Not stated	The BHUDRH is participating in a student-tracking study to monitor the longer-term career choices of students of all health disciplines, including those who went to Broken Hill for their rural placement. The potential to compare the career choices of students from different disciplines and different universities exposed to the Broken Hill program with students from the same faculties and universities placed elsewhere will allow for a more robust evaluation than would otherwise be possible

Article	Research Objectives	Study Design	Sample	Setting	Intervention-Education Design	Findings	Implications for Practice	Implications for Future Research
						With a strong academic team now in place, new units of study are being developed in collaboration with feeder universities, the first of which will offer elective placements for student nurses in remote health practice, primary health care and Indigenous health that will incorporate structured teaching and inter-professional learning that is locally developed and delivered		
Larsen, K., & Perkins, D. (2006). Training doctors in general practices: a review of the literature	Assess the evidence of whether new forms of medical training, where substantial training takes place in general practice, will be acceptable to General Practitioners (GPs)	Narrative literature review (systematic methods)	Characteristics of studies included in the review not specified within the article	Rural General Practitioner (GP) training	Training for medical students within General Practice settings	The key concern for GPs agreeing to accept training roles is their intrinsic motivation. Questions of infrastructure, financial and other extrinsic rewards are secondary	Medical schools must develop appropriate placement models, overcome obstacles faced by practices, and implement effective skills training, so that GPs can deliver major elements of the curriculum	Not stated
Lyle, D., & Kerr, C. (2001). Building capacity in rural health	Describes what effective and sustainable infrastructure is needed to build health workforce capacity in rural and remote areas	Commentary	Not applicable	Not applicable	Not applicable	University departments of rural health fill a gap by attracting experienced academics to work in the bush, and through those institutions provide educational opportunities and support to rural practitioners that were not previously available	Progress with capacity building in rural health will depend on encouraging a strong level of participation among rural health workers to look beyond the limits of their established activities and to engage in constructive discussion on improving capacity. In rural areas this has the potential to combine local expertise and networks to achieve greater capacity, self-reliance and sustainability of effort	Not stated

Article	Research Objectives	Study Design	Sample	Setting	Intervention-Education Design	Findings	Implications for Practice	Implications for Future Research
Humphreys, J. S., Lyle, D., Wakerman, J., Chalmers, E., Wilkinson, D., Walker, J., ... & Larson, A. (2000). Roles and activities of the Commonwealth Government University	Describe current activities of Australian University Departments of Rural Health (UDRHs)	Commentary	Australian University Departments of Rural Health (UDRHs) as of 2000	University Departments of Rural Health (UDRHs) at Broken Hill, Mount Isa, Shepparton, Launceston, Whyalla, Alice Springs, and Geraldton	Details education, research, and population health activities across UDRH campuses - see original article for further detail regarding these activities	Suggested that UDRHs are acting to increase the infrastructure and intellectual capital in key rural and remote regions, establish relevant teaching and support environments, enhance the opportunities for student placements in rural and remote settings, encourage experienced academic staff to spend time in the bush and foster coalitions of interest and partnerships across research and development organisations	There is an emerging academic discipline of remote health. Rural health has been recognised for some years now as a distinct discipline and has reached a level of maturity. In general, 'remote' has been lumped in with 'rural', without the distinguishing features of remote health practice clearly elucidated. The Centre has a specific focus on remote health issues. It provides an opportunity to tap in to the rich vein of remote-area experience and innovation in the Northern Territory and marry this with academic rigour, credibility and the intellectual resources of the universities	Not stated
